# Deciphering Desorption Pathways and Mechanisms of Peptide Supramolecular Structures Thermodynamically and Kinetically by High-Speed AFM

**DOI:** 10.1021/acscentsci.5c00215

**Published:** 2025-04-02

**Authors:** Linhao Sun, Jinhua Hu, Yurtsever Ayhan, Chen Chen

**Affiliations:** † 12858 WPI Nano Life Science Institute, Kanazawa University, Kakuma-machi, Kanazawa 920-1192, Japan; ‡ Division of Natural System, Graduate School of Natural Science and Technology, Kanazawa University, Kakuma-machi, Kanazawa 920-1192, Japan; § 542778 Earth-Life Science Institute, Institute of Future Science, Institute of Science Tokyo, Meguro-ku, Tokyo 152-8550, Japan

## Abstract

Studying molecule desorption from solids has attracted much interest in crude oil exploitation, self-cleaning nanotechnology, and biomedicine. Much work on polymer/biopolymer desorption has addressed the effect of pH, salts, and others on desorption features fitted by established models. However, molecular desorption pathways and mechanisms are still poorly understood due to lack of (i) a good model directly revealing nano- to microscale desorption characteristics and (ii) a powerful nanotool enabling the capture of every detail within sufficient spatiotemporal resolution. We utilized well-organized peptide supramolecular arrays (pSMAs) as a model system and high-speed AFM to investigate molecular desorption pathways and the mechanism in thermodynamics and kinetics. Temperature as a key parameter affects both peptide desorption and diffusion processes, leading to changes of pSMA coverage and ordered-to-disordered structures. Significantly, we found distinct desorption pathways of pSMAs in kinetics, including no desorption, one- or double-end desorption, and middle desorption of nanowires (NWs). Therefore, we proposed a “stop-to-activate” mechanism. Besides, the desorption characteristics of single NWs and pSMAs were well fitted by an exponential curve following classic desorption models. This work provides a good guideline for studying other molecules’ or assemblies’ desorption and sheds light on investigating drug effects on disassembly of amyloid protein fibrils in treating neurodegenerative diseases.

## Graphical abstract

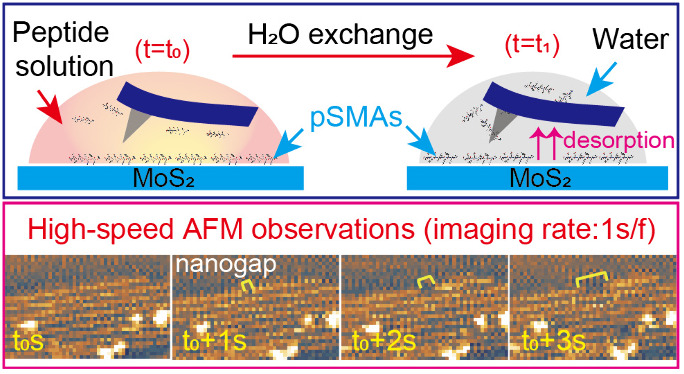

## Introduction

Molecular desorption, a reverse process of adsorption,^1–5^ from solid surfaces has been attracting much attention in the fields of crude oil exploit,^6,7^ self-cleaning nanotechnology,^8,9^ and biomedicine in drug-triggered degradation of neurodegenerative fibrils.^10,11^ Desorption behaviors of molecules including polymers,^12,13^ surfactants,^14^ proteins,^15^ DNAs,^16^ and other synthetic molecules^17^ at interfaces are highly complicated due to its characteristics dominated by not only thermodynamics but also kinetics. Temperature as a key parameter in thermodynamics and can induce changes in the desorption free energy, desorption time constant, exponent factor, and molecular diffusion coefficient. Also, time as a critical factor in kinetics affects a serial surface desorption characteristic such as attached molecule area on solids, desorption rates, and other properties. Besides, the molecular desorption process is accomplished in a short time scale, with most molecules being only a few nanometers in size.^18^ Thus, directly capturing and recording the surface characteristics and mechanism of molecular desorption in thermodynamics and kinetics with high spatiotemporal resolution is a big challenge. Therefore, to decipher the molecular-scale desorption characteristics and underlying mechanisms of molecules or assemblies on solids in kinetics and thermodynamics, we need to overcome two significant issues, one is a good model for studying molecules’ and assemblies’ desorption and another is a powerful nanotool with sufficient spatiotemporal resolution allowing for recording and capturing desorption details.

It is well-known that peptide self-assembly on solid substrates such as graphite and MoS_2_ surfaces through bottom-up techniques enable the formation of long-range ordered peptide supramolecular arrays (pSMAs) undergoing surface absorption and aggregation processes.^19–23^ The atomic force microscopy (AFM) technique has been utilized to probe the nanoscale morphology and structure of these assemblies, as well as their thermodynamics and kinetics.^20,22,24^ If we can controllably carry out an opposite process, that is, stimulating pSMA desorption, it would probably offer an opportunity to address desorption characteristics and mechanisms. Well-organized pSMAs are anchored atop the underlying substrates by noncovalent interaction and weak interpeptide interactions, making them particularly responsive to external stimuli that can trigger their desorption. As surveyed, most methods for affecting molecule–substrate interactions to induce polymer molecule desorption include pH and salts,^25^ organic solvents,^26^ or modulating molecule concentration gradients.^27^ Here, we employed a simple method by a water-exchange process, where the remaining peptide solution on solids was replaced by pure water. This method promotes a concentration gradient (CG) formation between peptides on solids and peptides in water. The CG as a driving force induces the occurrence of peptide desorption. Moreover, preformed pSMAs on solids provide a good contrast, allowing to directly reveal changes before and after peptide desorption such as reductions in pSMA length and area. Besides, to enhance spatiotemporal resolution for observations, we utilized high-speed AFM (HS-AFM) as a nanotool, with nanometer and milli-second resolutions as reported,^28–30^ to give insight into the unprecedented details of long-range ordered pSMAs.

Peptide desorption from pSMAs, unlike counterpart peptide adsorption in self-assembly, underwent an ordered phase to disordered phase transition, the energy landscape from low to high energy driven by an entropy increase in the system. In the desorption process, pSMAs on solids go from the initial equilibrium state to a second equilibrium state. Besides, degrees of freedom of peptides on solids in the pSMA desorption process are significantly different from that of the pSMA formation process via adsorption. The occurrence of local desorption is highly limited, such as peptide domain boundary or defects. Therefore, peptide desorption is not a simple reversible process of peptide adsorption widely studied in peptide self-assembly. Also, differing from polymer or biopolymer desorption in previous studies,^1,12,15,31^ most of the works have been focusing on molecular weight effects and desorption models such as exponent curve.^32,33^ Our current work is keen on desorption characteristics from pSMAs, specifically, peptide desorption pathways in the nanoscale regime transition to large scale, which are rarely explored. Through studying these desorption pathways, we also aim to provide insight into the unknown desorption mechanism of pSMAs.

In this work, we utilized *in situ* HS-AFM to investigate the pathways and mechanisms of pSMA desorption on MoS_2_ and graphite surfaces in terms of thermodynamics and kinetics. Temperature caused changes in pSMA coverage and structures from ordered phases to disordered phases. A “stop-to-activate” desorption mechanism was proposed, highlighting distinct local desorption pathways including no disassembly, unidirectional disassembly, asymmetrical bidirectional disassembly, and middle disassembly. Furthermore, single nanowires (NWs) desorption of pSMAs revealed two distinct exponent curves with factors of 0.67 and 1.1 while the desorption of entire pSMAs followed an exponential decay curve with a factor of 0.66, consistent with classic desorption models. Besides, the reassembly and phase transition of pSMAs in desorption kinetics were visualized. This work would provide a good guideline for studying the desorption characteristics and mechanisms of other molecules or molecular assemblies. It would also facilitate the studying of drug-induced decomposition of amyloid protein fibrils in treating neurodegenerative diseases such as Alzheimer’s and Parkinson’s diseases.

## Results and Discussion

### Peptide Sequence and 2D Arrays on MoS_2_ Surface

In this work, we utilized a peptide sequence, PKFKIIEFEP (named “FI”), which has been found to possess the capability of forming nanofibrils in solution.^34^ The FI peptides include MoS_2_-binding domains (F residue) and positively and negatively charged residues (K and E) (Figure 1a). Other basic information about the FI peptide was given (Figure S1). A topographic image (Figure 1b) showed the formation of long-range ordered FI pSMAs on the MoS_2_ surface by the self-assembly process. Three preferential orientations of pSMAs reflecting 3-fold symmetry of the underlying MoS_2_ lattice were observed by a fast Fourier transform (FFT) image. The specific orientation was further confirmed by a schematic model with an intersect angle of 60° (Figure 1c). The structural ordering of FI pSMAs was determined by a balance between peptide–MoS_2_ interactions and interpeptide interactions. The π electrons of the side chain phenol group of FI peptide can provide binding sites for interacting with MoS_2_ lattice.^35,36^ The interpeptide interactions are from electrostatic interactions via the negatively charged E residue and positively charged K residue of the FI peptide dimer (Figure 1d). We aimed to investigate the molecular scale desorption features of well-organized FI pSMAs in thermodynamics and kinetics by high-speed AFM. A schematic model (Figure 1e) depicted our methodology, which involved (i) formation of well-organized pSMAs, (ii) water incubation experiments, and (iii) AFM imaging to capture desorption process.

**Figure 1:**
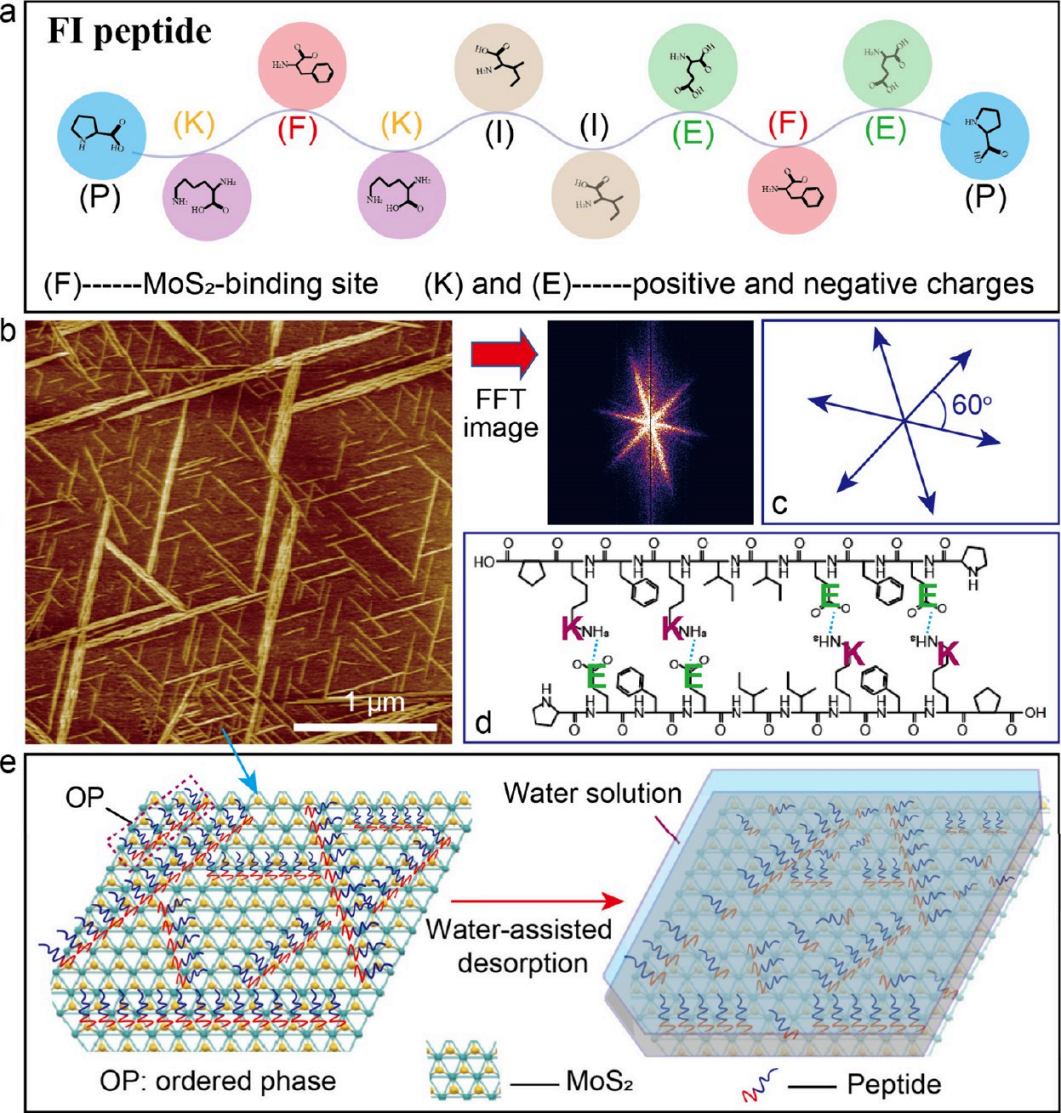
(a) Peptide sequence containing binding domains and charged domains. (b) An AFM image showing well-organized FI pSMAs and an FFT image indicating preferential orientations. (c) A schematic model depicting specific orientation of pSMAs with intersect angle of 60 degrees. (d) FI interpeptide interaction from electrostatic interaction of positively and negatively charged residues. (e) A schematic model of pSMAs desorption via water incubation experiment.

### pSMA Desorption Thermodynamics

We first investigated the thermodynamic features of FI pSMAs under water incubation conditions. A classic theoretical model has found that raising temperature can promote desorption process.^24,31^ Therefore, we examined pSMA desorption characteristics at temperatures of 25, 35, and 45 °C, respectively. A detailed experimental procedure was depicted (Figure 2a). Initially, 10 μM FI peptide solutions were incubated on freshly exfoliated bulk MoS_2_ surfaces for 1 h. The sample was dried by N_2_ blow and imaged by *ex situ* AFM. Then, water incubation was performed on the same sample for an expected time, followed by another N_2_ drying step and a second round of *ex situ* AFM observations. Through repeating the same procedure at each temperature, we checked morphological and structural changes of FI pSMAs as observed below. FI pSMAs formed an ordered phase (OP) and have a high coverage as initially prepared without water incubation (Figure 2b). After 60 min of water incubation, there still remained structural ordering, as confirmed by inset FFT images. As time extended over 90 min, structural ordering of pSMAs was broken. However, at both 35 and 45 °C, the initial coverage of FI pSMAs before water incubation was relatively lower compared to 25 °C, and the structural order of the assemblies was less pronounced (Figures 2c and 2d). Prolonged water incubation not only further disrupted the structural order but also accelerated the desorption process of the pSMAs. For example, after 150 min of incubation at 35 °C, the ordered pSMAs were largely replaced by an amorphous phase (AP) of peptide, indicating significant desorption and loss of structural integrity.

**Figure 2:**
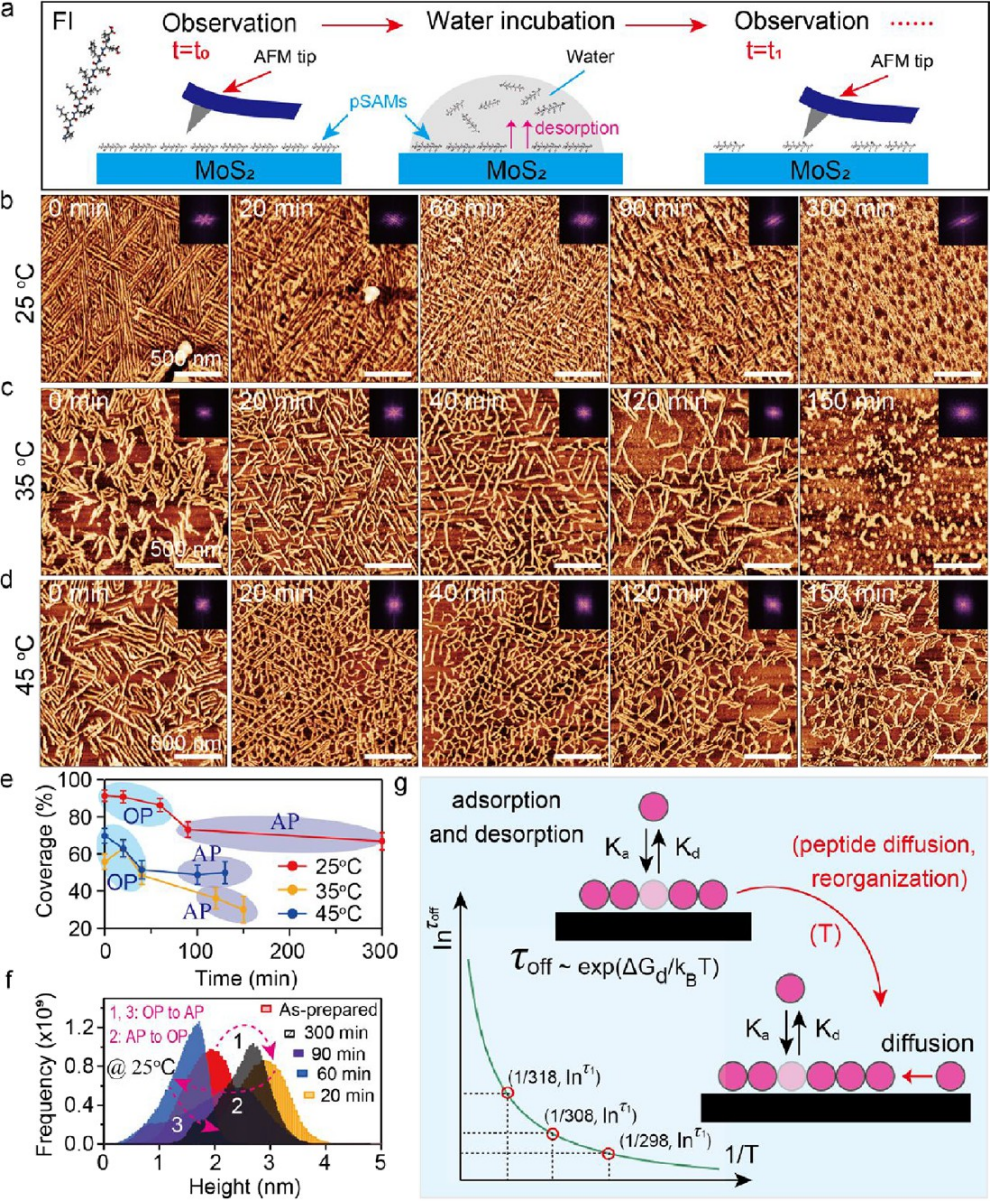
Morphologies and structure changes of FI pSMA desorption in thermodynamics under water condition. (a) Experimental procedure. (b–d) Topographic images of pSMAs under 25, 35, and 45 °C with different water incubation times, respectively. (e) Coverage and phase changes of pSMAs with water incubation time. (f) Quantifying structural changes of pSMAs. (g) A schematic displaying temperature effect on not only desorption but also diffusion and reorganization.

To quantify the thermodynamic desorption features, we further analyzed peptide coverage changes over water incubation time, as plotted (Figure 2e). We found the following features. (a) A pronounced reduction in OP coverage was observed with rising incubation temperature (blue color). (b) The rate of peptide coverage reduction became less prominent with extending water incubation time. For example, at 25 and 45 °C, an almost plateau feature of AP coverage was observed. (c) Peptide coverage showed no positive correlation with temperature change during the water incubation process. As found, peptide coverage at 45 °C was higher than that at 35 °C. The high temperature, on one hand, promotes the desorption of peptide molecules. On the other hand, it facilitates peptide diffusion and rearrangement to form new pSMAs. The trade-off between them could determine the final peptide coverage. Indeed, we have found a slight increase of peptide coverage during water incubation time *t* = 20 min at 35 °C in contrast to as prepared sample, which further supports the above explanation. Besides, statistical analysis of pSMA heights indicated that the reversible OP (AP) to AP (OP) transition induced by a long water incubation time led to a height increase (decrease) since the folded structure of well-organized pSMAs was partially disrupted (Figure 2f and Figure S2). To further understand the correlation between coverage changes and varying temperature, we employed a classic theoretical model^1^ to explain pSMA desorption as eq 1:

1 $$\tau_{off}\approx \exp\left(\Delta G/k_{B}T\right)$$

where τ_off_ is the desorption time constant; Δ*G* is the free-energy change upon desorption; *k*_B_ is the Boltzmann constant; and *T* is the absolute temperature. The rate of desorption is supposed to be proportional to the surface coverage, θ­(*t*), which leads to exponential decay, as discussed in the latter kinetic section. From an inverse correlation between τ_off_ and temperature *T* in eq 1 as well as the plot (Figure 2g), high temperature induces a short desorption time constant, indicating a fast desorption rate. If considering the same incubation time, pSMA coverage on the substrate should dramatically reduce at high temperature such as θ (25 °C) > θ (35 °C) > θ (45 °C). Our observation gave an opposite correlation at 35 and 45 °C, where θ (35 °C) < θ (45 °C) (Figure 2e). The reason is that a high temperature probably promotes the surface diffusion and rearrangement of peptides, leading to the formation of new pSMAs on MoS_2_ surfaces. The rearrangement of desorbed peptides still partially followed a certain crystallographic orientation on MoS_2_ via a “lattice matching” mechanism as widely found.^21,22,37^

### pSMA Desorption Kinetics

In previous temperature-induced desorption results, at a temperature of 25 °C, FI peptides can maintain highly ordered structures on MoS_2_ surfaces for a relatively long time. A system with a long-term ordered phase allows to continuously capture and record the desorption features, intrinsic pathways, and mechanisms. Therefore, in later sections, our kinetic observations of peptide desorption were conducted at a temperature of 25 °C. Then, we investigated the desorption kinetics of pSMAs by an *in situ* HS-AFM technique, which provides molecular resolution and subsecond time resolution,^28^ allowing for insight into the momentary intrinsic desorption characteristics and mechanisms. The procedure for sample preparation and imaging was drawn in a schematic (Figure 3a), with additional details provided in the experimental section. Unlike the drop-cast experiments by *ex situ* AFM observations (Figures 2b–2d), *in situ* HS-AFM enables the visualization of many detached freely diffused peptide nanoclusters as white dots in a water-exchanged environment (Figure 3b). Some of these nanoclusters appeared at one or both ends of single pSMAs. We zoomed in at one location as marked by the blue square; HS-AFM provided desorption details at a local site labeled by two colorful dash circles. Red and white circles indicated NWs of pSMAs with and without changes as the imaging time increased, respectively. The corresponding magnified images (Figure 3b, time-lapse images) revealed the formation of a tiny nanogap (∼6 nm) in the middle of single pSMAs at *t* = 38 s. The nanogap remained unchanged from *t* = 41 to 45 s, after which its size gradually increased until a whole single NW of pSMAs fully desorbed at *t* = 54 s (Figure S3). Besides, we noticed that desorption rates at two ends of single NWs of pSMAs were asymmetrical (Figure S4).

**Figure 3:**
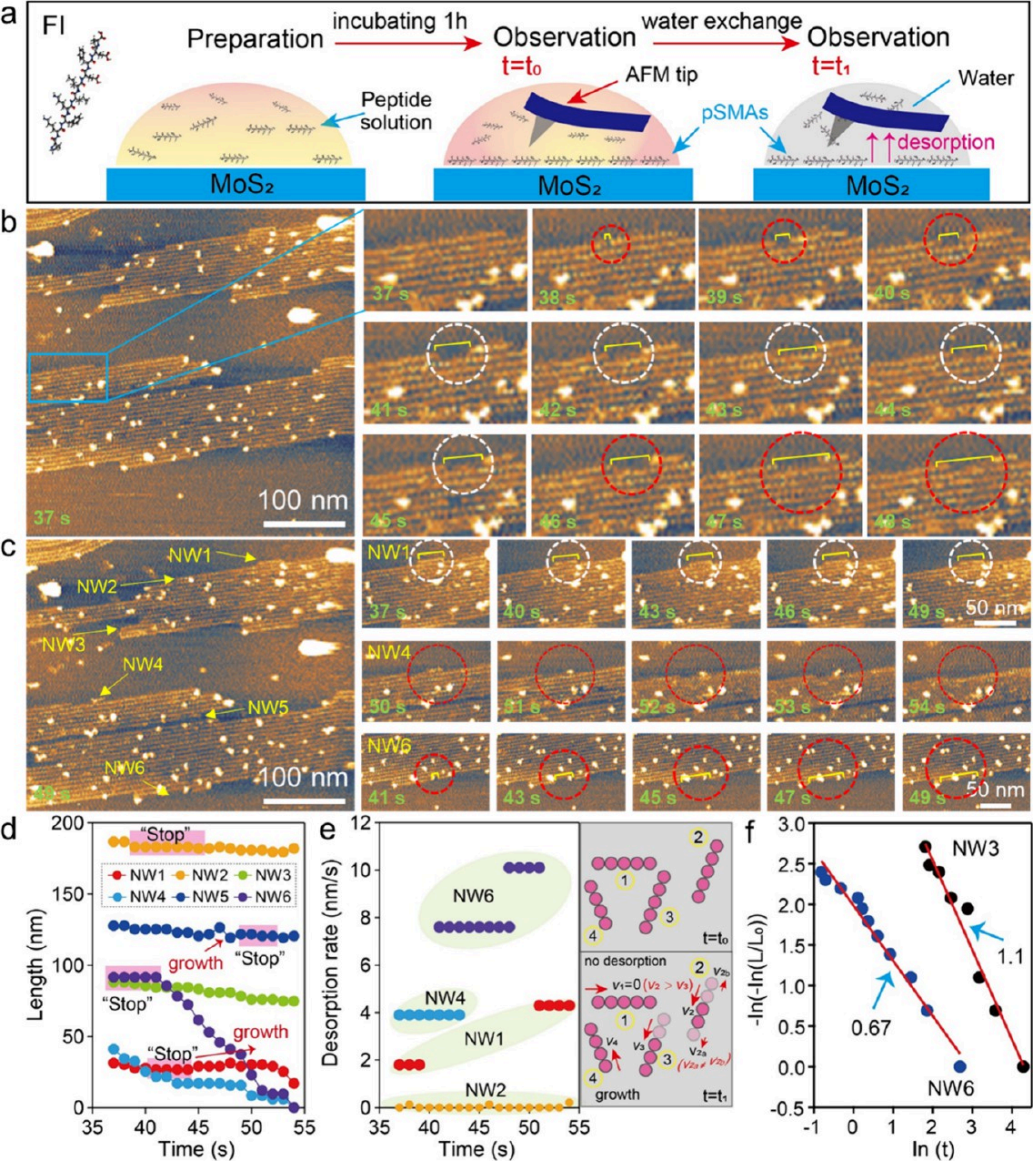
*In situ* HS-AFM recording FI pSMAs desorption in kinetics with molecular resolution. (a) Experimental procedure. (b and c) Real-time observations of FI pSMAs disassembly in water condition, with magnified images revealing the different desorption pathways in kinetics. (d and e) Quantifying the desorption characteristics, including pSMAs length and disassembly rates. (f) Two distinct desorption modes of pSMAs in water condition.

To further quantify the surface characteristics of pSMAs desorption kinetics, we analyzed the time-dependent length changes of six selected NWs. The results (Figure 3c) indicated desorption heterogeneities among these NWs. In the case of NW1, the length of NW1 almost has no noticeable changes, indicating NW1 in a “stop” or say “dormant” state. Such “stop” state was also found in other NWs in a short time scale (Figure 3d, pink shadows). As the scanning time in water solution was extended, pSMAs desorption was activated and gradually shortened their lengths such as NW4 (Figure S5). Besides, a reversible process, that is, peptide growth (Figure S6, white squares), took place accompanying pSMAs desorption kinetics. Such phenomena in the above experimental observations were also captured in other sets of experiments (Figure S7). Based on these findings, we proposed a “stop-to-activate” desorption mechanism for pSMAs. We also quantified the desorption rates of NWs 1, 2, 4, and 6 in a range from 0 to 10 nm/s (Figure 3e). Distinct desorption rates were observed at different time intervals, such as for NWs 1 and 6. Considering typical interpeptide periodicities of 0.5 to 1 nm along the pSMA direction,^20,22,23^ our quantitative analysis suggested that up to 20 single peptide units could be disassembled within 1 s. Such a desorption rate is comparable to the HS-AFM imaging rate, providing a molecular scale understanding of pSMA desorption. Here, a schematic model was drawn to reveal the key features of pSMAs desorption including no desorption, heterogeneous desorption, and short-term growth during the desorption process (Figure 3e, a schematic in rightmost).

In the literature, desorption kinetics of polymers or biopolymers on the surface typically have shown exponential kinetics or exponential with index one-half depending on temperatures.^1^ Distinct from disordered structures of these molecules, pSMAs are highly ordered on the MoS_2_ lattice. Therefore, we also investigated their desorption kinetics; specifically, we looked into each NW’s desorption dynamics. The desorption rate is time-dependent because of the intermittency of surface detachment, represented below in eq 2:

2 $$\frac{d\theta \left(t\right)}{dt}=-R\left(t\right)\theta \left(t\right)$$

where *R*(*t*) is the steady-state flux per unit concentration from a planar surface, defined by eq 3. *D* is the diffusion coefficient of peptide; β is the exponent factor.

3 $$R\left(t\right)=\left(D/\pi t^{\beta }\right)$$

upon integration of eq 2 and eq 3, we obtained the following important eq 4:

4 $$\frac{\theta \left(t\right)}{\theta_{0}}\approx \exp\left[-(t/{\tau_{off)}}^{\beta }\right]$$

where θ_0_ is initial pSMA coverage adsorbed on the MoS_2_ surface at *t* = 0. Since well-organized pSMAs on substrates typically have fixed periodicities along the NW direction and lateral direction,^20,23^ a unit cell of pSMAs has a same size. Therefore, we can utilize NW length by replacing coverage to calculate a single NW’s desorption kinetics, as described by eq 5:

5 $$\frac{L\left(t\right)}{L_{0}}\approx \exp\left[-(t/{\tau_{off)}}^{\beta }\right]$$

Taking NW3 and NW6 as examples, based on eq 5, we obtained two distinct exponent factors of 0.67 and 1.1 for NW6 and NW3 (Figure 3f and Tables S1 and S2). The values of 0.67 and 1.1 are close to typical values of 0.5 and 1.0, as widely found in polymer/biopolymer desorption at temperatures below 25 °C and at high temperatures, respectively.^1,13,31^ Therefore, single NWs from pSMAs displayed two distinct desorption models under water conditions.

### Insight into Desorption Details of pSMAs in Kinetics

Regarding the results by *in situ* HS-AFM observations, we further looked into more details in pSMAs desorption kinetics. For example, nanoclusters attached to one or both ends (Figures 4a and 4b, yellow circles), blocking pSMAs desorption (Figures S8 and S9). Also, two different phases of FI peptides followed the same desorption behaviors (Figure 4b, long white and blue arrows at *t* = 0 s). Such surface characteristics of pSMA desorption were also found along other crystallographic orientations (Figure 4c). These nanoclusters showed a strong stability in water condition (blue, yellow, and white arrows). Occasionally, we observed random coil-like structures attached to nanoclusters (Figure 4c, a magnified image, Figure S10), indicating a gradual disassembly of nanoclusters. Once nanoclusters were removed, pSMAs were activated and commenced desorption in a row-by-row manner (Figure S11, red circles). A quantitative analysis of these nanoclusters showed heights of 2 ± 0.3 nm, doubling the height of well-organized pSMAs (Figure 4d and Figure S12). The sizes of these nanoclusters were centered at 7.5 nm (Figure 4e, a histogram), which is comparable to the periodicity of 5.7 nm along the pSMA lateral direction (Figure 4e, a FFT image). Based on these parameters, these nanoclusters were composed of several single peptides. Besides, a comparison of desorption rates along pSMAs and lateral directions (*v*_a_ vs *v*_b_) showed significant differences. Peptide desorption rate along the pSMAs direction is much faster than that along the lateral direction. It is worth noticing that the desorption process could be commenced in the certain middle location of single pSMAs as one or both ends of pSMAs were blocked by nanoclusters (Figure 4c, yellow arrows).

**Figure 4:**
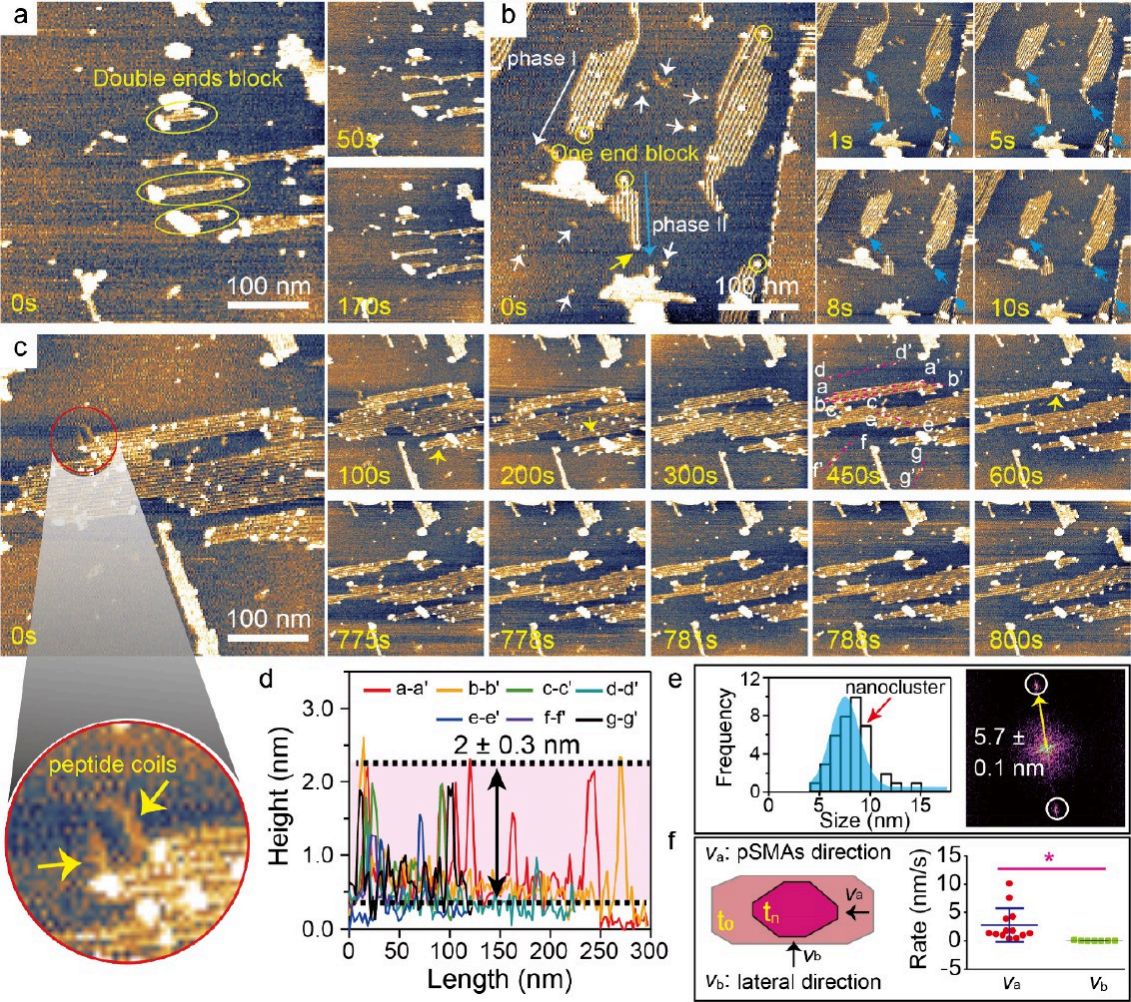
Dominant and activated states of FI pSMAs desorption. (a) Dominant state of pSMAs via double end block of nanoclusters. (b) Dominant state of pSMAs via one end block of nanoclusters. (c) Activated state of pSMAs taking place in a certain location of pSMAs; a magnified image showing coil-like single peptide attached to nanoclusters. (d) Heights of nanoclusters. (e) Size distribution of nanoclusters comparable with a lateral periodicity of pSMAs revealed by an inset FFT image. (f) Desorption rates along NW and lateral directions, respectively.

### Phase Transition of pSMAs in Desorption Kinetics

In our HS-AFM observations, we also noticed that the phase transition of FI pSMAs took place in desorption kinetics. Prior to imaging in water condition, *in situ* HS-AFM results (Figure 5a) showed two well-organized phases, phases I and II (*t* = 0 s, blue and purple arrows), of pSMAs on the bulk MoS_2_ surface. The observations of the two phases were consistent with the results shown (Figure 4b). These two phases have an intersect angle of 30°, confirmed by a crystallographic orientation model (Figure 5b). As scanning time was extended, the size of phase II gradually grew larger (purple color). Oppositely, the size of phase I was noticeably reduced and isolated into several small domains (Figure 5c, white arrows). To have a better understanding of the above phenomenon, we further quantified the coverage changes of each phase. A plot displayed that coverages of both phases I and II of pSMAs underwent a dormant state initially (Figure 5d, blue shadows), where their coverages had no significant changes (Figure S13). Then, phase transition between phases I and II was commenced, although the initial size of phase II was much smaller than that of phase I. This result is distinct from the traditional “Ostwald ripening” phenomenon,^38,39^ where a large size of grains grows at the expense of small domain sizes. The above results may suggest that phase II is a thermodynamically stable phase while phase I is an intermediate phase, where a lattice matching mechanism between a unit cell of pSMAs and the underlying solid lattices needs to be satisfied as addressed.^37,40^ Once the phase transition of the two phases was ended, phase II further underwent desorption kinetics with gradually reduced coverage (Figure 5d, red shadow, Figure S14). Finally, we also plotted a correlation of pSMAs coverage and scanning time following eq 4, providing an exponent factor of 0.66 (Figure 5e and Table S3), which is similar to the previous results of single NW desorption analysis (Figure 3f).

**Figure 5:**
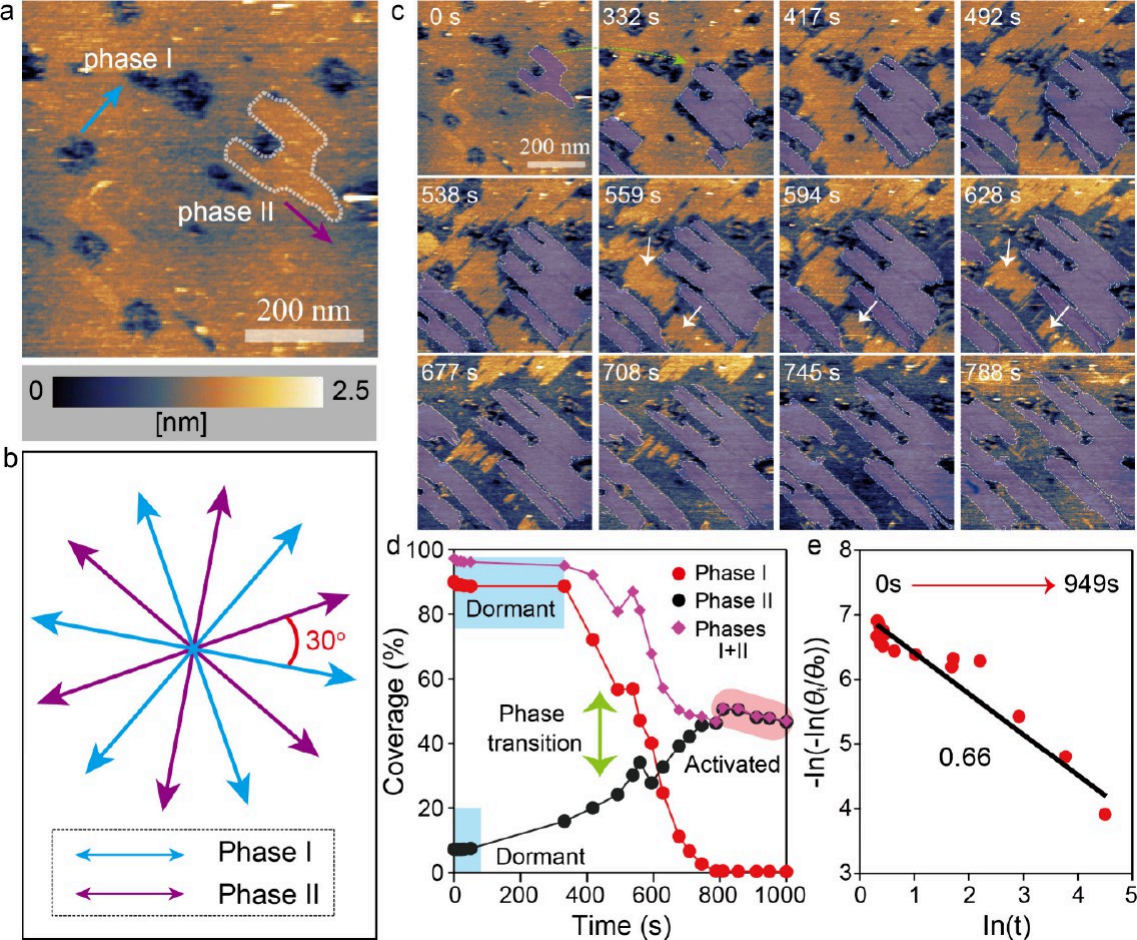
Phase transition and kinetic model in the FI pSMAs desorption process. (a) *In situ* HS-AFM revealing phase changes of pSMAs in desorption kinetics. (b) Orientation angle correlation between phases I and II. (c) Time lapse *in situ* HS-AFM topographic images showing dynamic changes of two phases. (d) Quantitative analysis for coverage changes with time of phases I, II, and I + II, indicating a competing interaction between phases I and II, and a reduced total coverage. (e) A kinetic desorption model of pSMAs including phases I and II with an exponent factor of 0.66.

### Desorption Pathways and Mechanism of pSMAs

To understand the desorption pathways and mechanisms of pSMAs in thermodynamics and kinetics, we draw schematic models revealing various desorption modes. The structural ordering of peptides on the MoS_2_ surface in a single row is governed by two key interactions, that is, peptide–MoS_2_ interaction (f_1_) and interpeptide interaction (f_2_). For multiple rows of pSMAs, lateral interpeptide interactions also need to be considered. In the later section, we mainly discuss pSMA disassembly in a single row. pSMAs underwent a “stop-to-activate” desorption mechanism. The binding affinity experiments showed that FI peptides have a binding constant of 1.03 μM^–1^ on the MoS_2_ surface (Figure S15). The π electron of Phe (F) could interact with the MoS_2_ lattice leading to peptides anchoring onto the MoS_2_ surface as reported.^36^ Two positively charged Lys (K) amino acids and two negatively charged Glu (E) amino acids were observed (Figure 1a). Interpeptides could interact with each other by electrostatic interactions between K and E amino acids to form a dimer-like unit (Figure S16). In case (i), no pSMAs disassembly took place as both ends of NWs were in a dormant state, where the disassembly at each end was blocked by the peptide’s aggregations (Figure 4a). The removal of these aggregations activated the desorption process, initiating unidirectional or bidirectional disassembly (cases ii and iii). The disassembly rates at each end of pSMAs depended on the surrounding physicochemical environments. A single peptide at each terminal needs to overcome the binding energy barrier from both interactions of f_1_ and f_2_. The desorption of pSMAs would create an energy increase although the remaining pSMAs were shortened and still retained structural ordering. The system entropy was increased by creating freely diffused peptides on MoS_2_ surfaces (Figures 3 and 4) and into water solution. Besides, in our experiments, as both ends of single pSMAs were blocked, a disassembly in a certain middle location of pSMAs took place, presented as case (iv). Differing from cases (ii) and (iii), single peptide desorption needs to overcome twice as much interpeptide interaction from both left and right neighboring peptides, which significantly increases the energy scale. The energy landscape in each case was drawn (Figure 6b). No disassembly in case (i) is energy minimum, where pSMAs are highly structural ordering. Additionally, as there maintained a balance between peptides in solution and peptides on the MoS_2_ surface, the whole system is in a metastable condition exhibiting a plateau feature (Figure 6b, an inset model).

**Figure 6:**
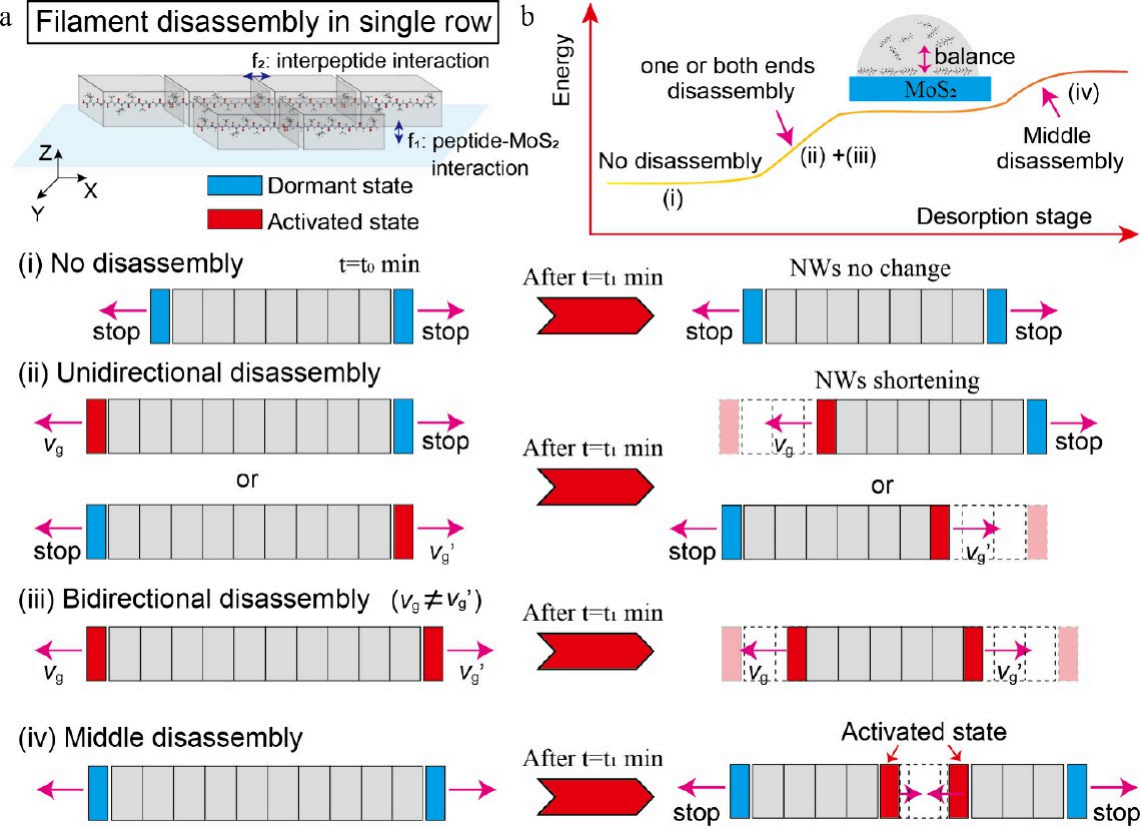
Desorption mechanism and characteristics of pSMAs. (a) Desorption modes of pSMAs at dormant and activated states including peptide–MoS_2_ and interpeptide interactions. (b) Energy landscape in the peptide desorption process, where a whole system changes from structural ordering to disordering, leading to enhanced energy states.

### pSMA Desorption Kinetics on Graphite Surface

Finally, to further confirm the above desorption pathways and mechanism, we examined the same desorption experiment of FI pSMAs on a freshly cleaved graphite surface by *in situ* HS-AFM measurements. The results (Figure 7a) showed the same desorption characteristics as found on MoS_2_ substrates including (i) no disassembly quantified by height profiles in Figure 7b, (ii) and (iii) one end or both end desorption shown by length reductions in Figure 7c, and (iv) middle desorption revealed by height profiles in Figure 7d. This result further supported our proposed desorption pathways for pSMAs on solid surfaces. Also, we have found that the desorption process of FI peptides on HOPG refers to two phases with an intersect angle of 30° or 90° (Figure S17), which is similar to that of FI peptides on the MoS_2_ surface.

**Figure 7:**
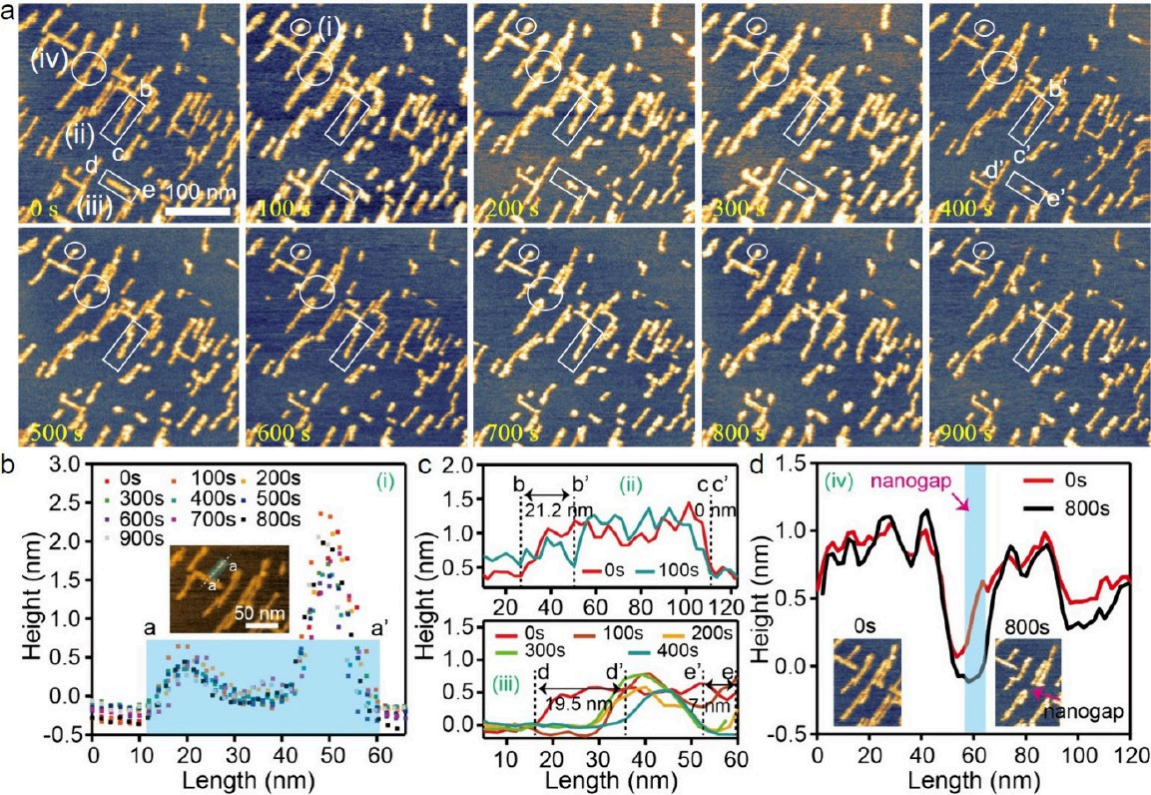
Desorption dynamics of FI pSMAs on graphite surfaces in water conditions. (a) Selected topographic images revealed by *in situ* HS-AFM measurements with scanning rate of 1 s/f. (b–d) Quantitative analysis of pSMAs desorption pathways including (i) no disassembly, (ii) one-end disassembly, (iii) double-end disassembly with asymmetrical desorption rates, and (iv) middle disassembly.

## Conclusion

We reported the molecular scale surface characteristics and mechanism of pSMA desorption from solids in thermodynamic and kinetic aspects by *in situ* HS-AFM observations. The morphological and structural changes of pSMAs via water incubation experiments at different temperatures were deeply investigated. Temperature can speed up quick peptide desorption while facilitating pSMAs rearrangement, leading to a nonmonotonic variation. Also, distinct pathways in desorption kinetics including (i) no disassembly, (ii) and (iii) unidirectional and bidirectional disassembly, and (iv) middle disassembly were revealed. We proposed a “stop-to-activate” desorption model. Desorption behaviors of single NWs and pSMAs meet a classic exponential curve model. Moreover, peptide phase transition and reassembly took place during the kinetic desorption process. This work sheds light on investigating a drug effect on disassembly of amyloid protein fibrils in caring for neurodegenerative diseases such as Alzheimer’s or Parkinson’s diseases.^41,42^

## Methods

### Materials

In this work, fresh bulk MoS_2_ and graphite by mechanical exfoliation were utilized as substrates for observations of the pSMAs desorption mechanism and characteristics. Peptide powders (purities >95%) were provided by GL Biochem (Shanghai), Ltd. The molecular weights and purities of each peptide were confirmed by mass spectrometry and high-performance liquid chromatography measurements, respectively.

### Sample Preparation

The FI peptide solutions with concentrations of 100 μM were prepared through dissolving several amounts of peptide powders into pure water (Millipore Corp.,18 MΩ cm at 25 °C). The prepared solutions were stored at −80 °C prior to AFM imaging.

### *Ex Situ* AFM Observations

Prior to AFM observations, the stock peptide solutions were warmed to room temperature, and then, 120 μL of FI peptide solution with concentrations of 10 μM was dropped onto freshly cleaved bulk MoS_2_ surfaces. The sample preparation was conducted in a humidity-controllable chamber for 1 h at room temperature. Then, samples were dried by a gentle nitrogen blow. Next, water incubation experiments were performed, the above samples were further incubated in a pure water condition for expected times, such as 20 and 40 min, and after drying, the samples were imaged by AFM measurements. In each experiment condition, self-assembled morphologies of peptides were characterized by a commercial AFM (Oxford Instruments, Asylum Research) in AC mode. The characterization was performed using a silicon cantilever with a resonance frequency of 300 ± 100 kHz and a spring constant of 26 N/m. The scanning speed was around 1.5∼3 Hz. The tip radius was 10 ± 2 nm.

### *In Situ* AFM Observation

First, 120 μL of FI peptide solution with concentrations of 10 μM was dropped onto freshly cleaved bulk MoS_2_ and graphite surfaces, followed by waiting for 1 h incubation. Then, the peptide solution was replaced by water with same volume. Immediately, *in situ* HS-AFM imaging (image rate: 1 s/f) was conducted by a homemade system in water environment. The topographic images, desorption dynamics, and phase transition dynamics of the FI peptide were captured using 160AC-NG cantilevers purchased from OPUS with a nominal spring constant of 26 N/m, a nominal tip radius of 8 nm, and a resonance frequency of 1.6 MHz.

### Coverage Calculation

As described in our previous work,^21,23,43^ we utilized image processing software (Gwyddion) for analyzing pSMAs coverage derived from the AFM topographic image. First, we fixed the tilt issue of surfaces from the obtained frames and set the height of the substrate to be 0 nm. To obtain peptide coverage, height information on peptide nanostructures from the topographic image was converted into a histogram, which was subsequently fitted using a Gaussian model by commercially available software, Igor (WaveMetrics, Inc., U.S.A.). A peak at the lowest position was attributed to the height of the substrate, while peaks having much larger heights represent the height distribution of peptide structures. An area corresponding to each fitting peak was obtained. Then, the ratio between areas covered by peptides and total area equals coverage.

### AFM Data Analysis

The AFM topographic images were treated by commercial software (WSxM 5.0 Develop 10.2). The coverages of self-assembled peptides were determined by using Gwyddion SPM data analysis software.

### Statistical Analysis

The histogram data of Figure 4e was obtained by using Igor software. The data of Figure 4f were analyzed using an unpaired *t* test and presented as the means ± s.d. *p*-values of <0.05 were considered to indicate statistical significance.

## Supplementary Material





## Data Availability

The data that support the findings of this study are available in the Supporting Information of this article

## Notes

The peptide information, pSMAs heights, HS-AFM images indicating desorption kinetics including different pathways, binding affinity, high-resolution AFM images, and tables for obtaining desorption exponent curves (PDF)
Transparent Peer Review report available (PDF)
