# Supplementary material for: Deciphering Desorption Pathways and Mechanisms of Peptide Supramolecular Structures Thermodynamically and Kinetically by High-Speed AFM
Source: ACS Cent Sci. 2025 Apr 2;11(5):672–82. doi: 10.1021/acscentsci.5c00215 (PMC12123461; doi:10.1021/acscentsci.5c00215)
Supplement: Supplementary file 1 [file oc5c00215_si_001.pdf]

Supporting information

Deciphering Desorption Pathways and Mechanism of Peptide Supramolecular Structures Thermodynamically and Kinetically by High-Speed AFM

Linhao Sun[1,*], Jinhua Hu[2], Yurtsever Ayhan[1], Chen Chen[3,*]

[1]WPI Nano Life Science Institute, Kanazawa University, Kakuma-machi, Kanazawa 920-1192, Japan

[2]Division of Natural System, Graduate School of Natural Science and Technology, Kanazawa University, Kakuma-machi, Kanazawa 920-1192, Japan

[3]Earth-Life Science Institute, Institute of Future Science, Institute of Science Tokyo, Meguro-ku, Tokyo 152-8550, Japan

*Corresponding authors

Email: sunlinhao0502@se.kanazawa-u.ac.jp; chenchen@elsi.jp

Table of contents

Sample characterization--2

Peptide information--2

pSMAs height changes--2

Desorption kinetics of single NWs--3

Asymmetrical desorption characteristics of single NWs--3

“Stop-to-activated” growth mechanism---4

pSMAs growth --5

peptide desorption features from other samples--5

Double ends block of pSMAs--6

One end block of pSMAs---6

Coil nanostructure of nanocluster as blocking end of pSMAs--7

Dormant state to activated state of pSMAs--7

Heights of pSMAs--8

Phase transition (dormant state) ---8

Desorption kinetics of phase II after phase transition--9

Binding affinity of peptides and MoS_2--9

Supplementary Table 1---10

Supplementary Tables 2--10

Supplementary Tables 3--11

Sample characterization

peptide information

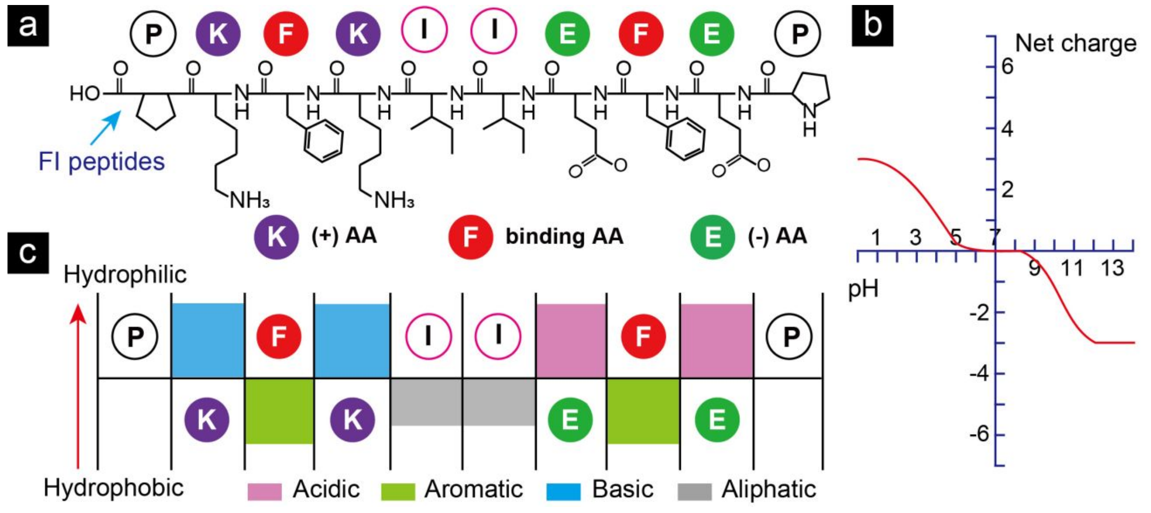

Figure S1. Basic information of FI peptide including (a) peptide sequence, (b) Hydropathy of each amino acid, (c) Net charge of FI peptide.

pSMAs height changes 10 μM peptide solution was placed onto freshly exfoliated MoS_2 surfaces and incubated for 1 hour. After gentle N_2 blowing to remove the remaining solution, the samples were immediately imaged by ex-situ AFM measurements. The height histograms indicated the effect of water incubation time on pSMAs structures.

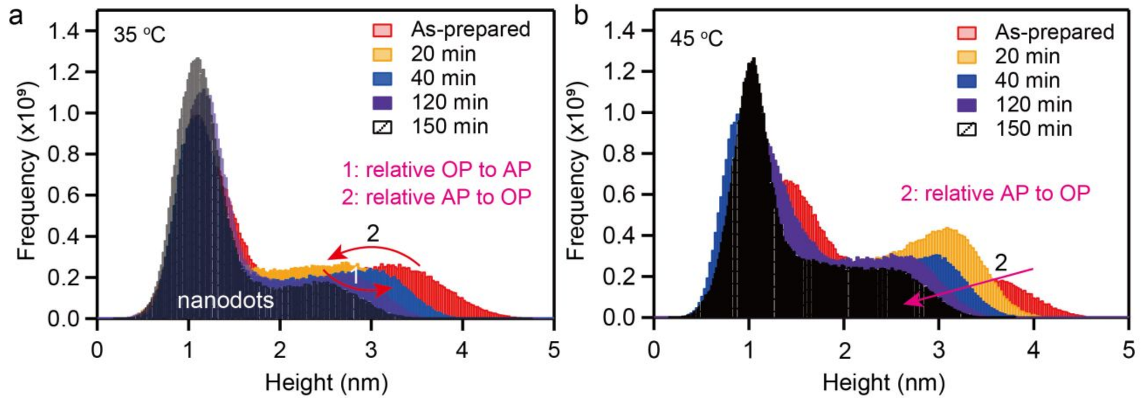

Figure S2. Height histograms of pSMAs measured by ex-situ AFM in dry condition showing structural changes as raising water incubation time.

Desorption kinetics of single NWs

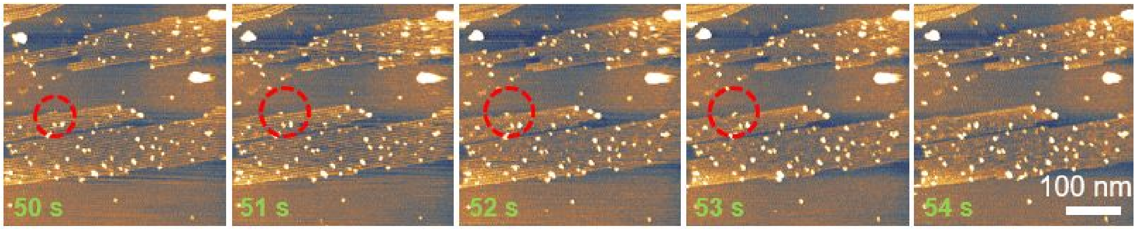

Figure S3. Desorption kinetics of single NWs revealed by HS-AFM measurements. Single NWs at the location marked by red dashed circles was gradually shorten until completely disappeared at t=54s.

HS-AFM observations revealing an asymmetrical desorption rates at both ends of single nanowires

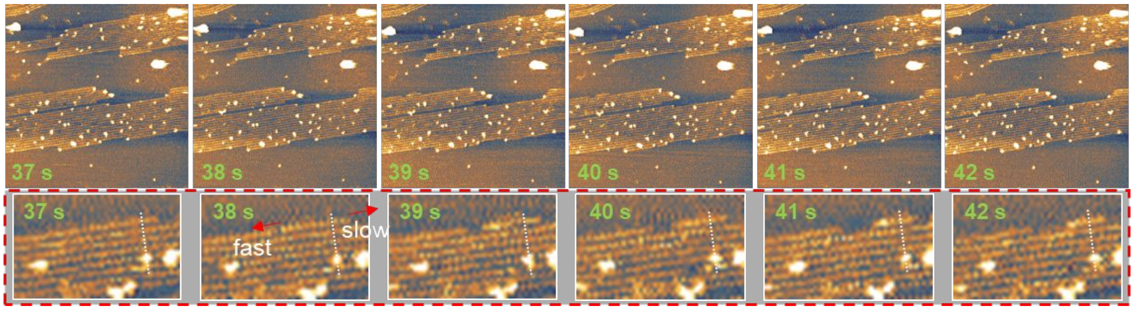

Figure S4. Asymmetrical desorption rates at both ends of single nanowires captured by HS-AFM observations. Red arrows indicating peptide desorption along both ends of a same nanowire. Dashed lines are utilized as a landmark to show the changes of single nanowires at both ends.

Height profile indicating a "Stop-to-activated" growth mechanism

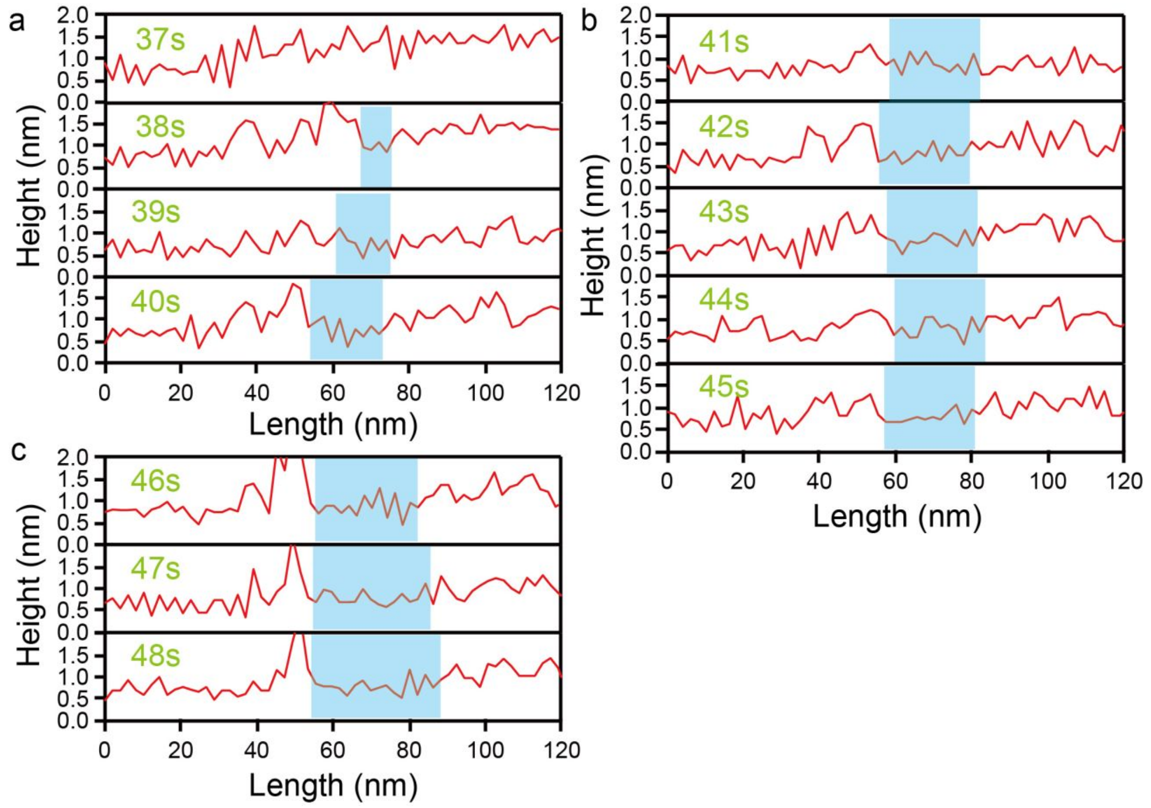

Figure S5. Quantitative analysis of nanogap length changes with scanning time of (a) 37s to 40s (nanogap enlarged), (b) 41s to 45s (no change), and 46s to 48s (nanogap enlarged).

pSMAs growth 10 μM peptide solution was placed onto freshly exfoliated MoS_2 surface. After water exchanging, the samples were immediately imaged by HS-AFM in water solution.

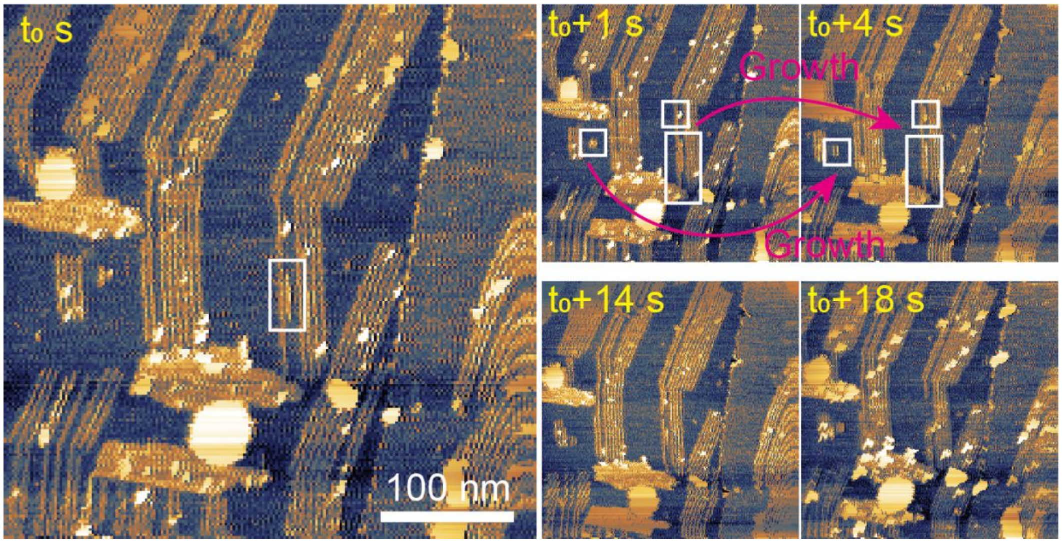

Figure S6. pSMAs growth labelled by white squares during desorption kinetics visualized by in situ HS-AFM measurements.

Peptide desorption features from other sample set

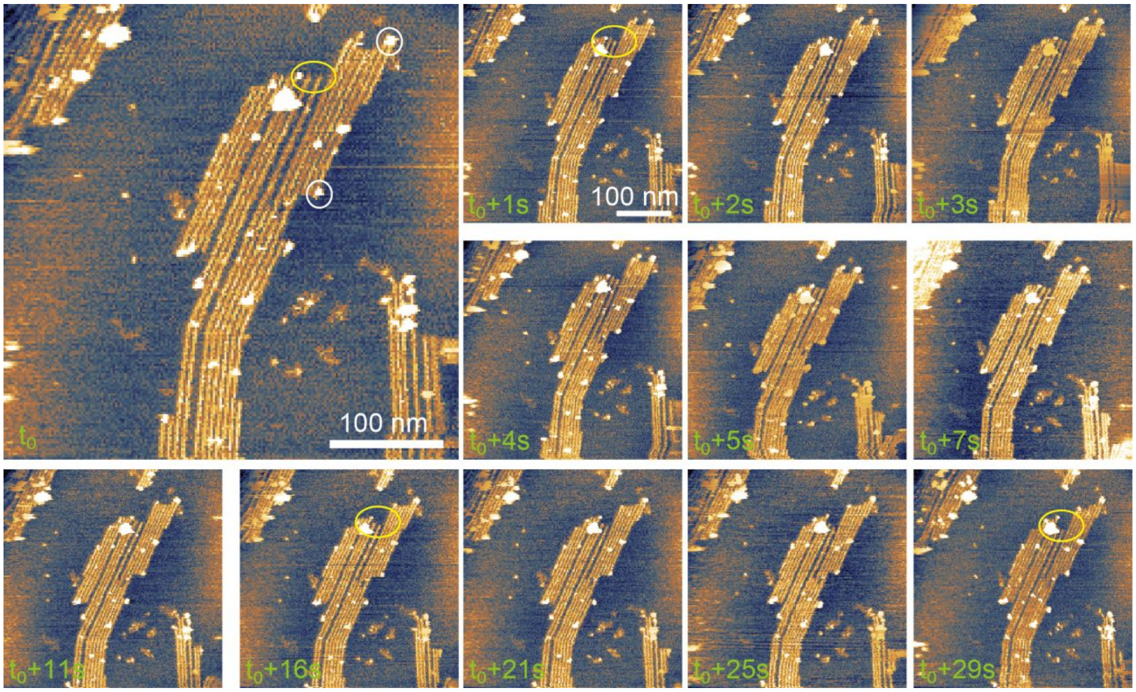

Figure S7. HS-AFM revealing same desorption pathways of pSMAs as found in Figure 3b-c.

Double ends block of pSMAs We observed that both ends of some NWs of pSMAs were attached by nanoclusters, which significantly delayed pSMAs desorption kinetics. HS-AFM imaging rate was 1 f/s, image pixels: 200 by 200.

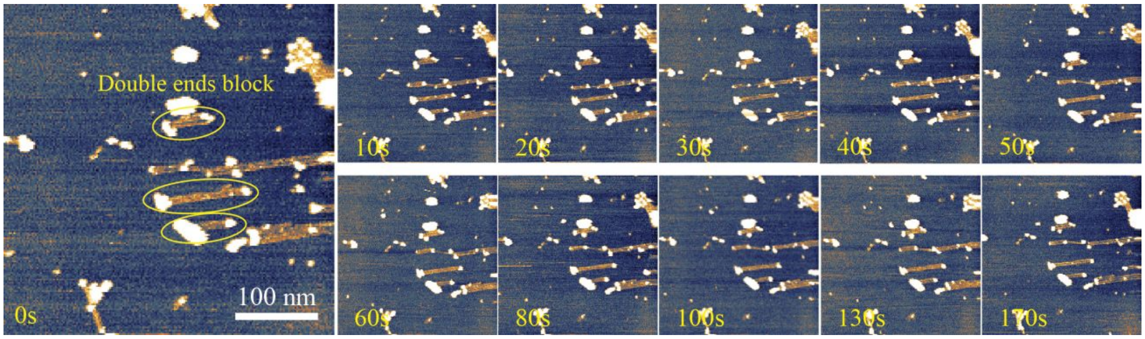

Figure S8. *In-situ* HS-AFM observations revealing no growth of pSMAs as double ends were blocked by nanoclusters, indicating a high stability of these nanoclusters (see yellow circles).

One end block of pSMAs We observed that one end of some NWs of pSMAs were attached by nanoclusters, which also significantly delayed pSMAs desorption kinetics. HS-AFM imaging rate was 1 f/s, image pixels: 200 by 200.

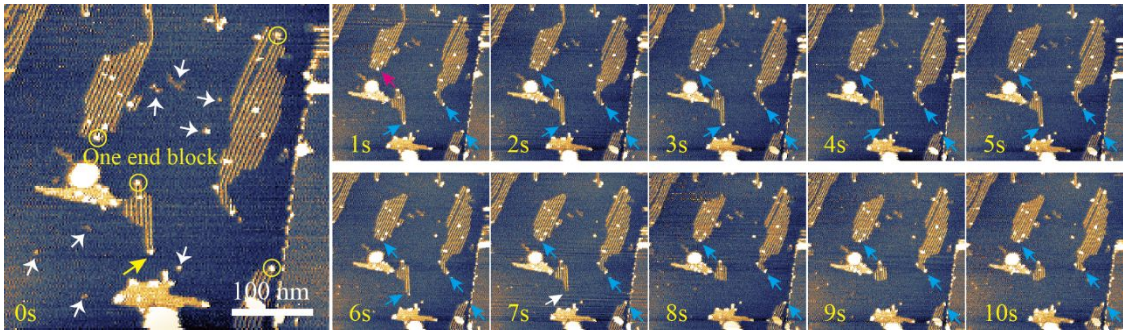

Figure S9. *In-situ* HS-AFM observations revealing no desorption of pSMAs as one of two ends was blocked by nanoclusters (see blue arrows).

Coil nanostructure of nanocluster as blocking end of pSMAs

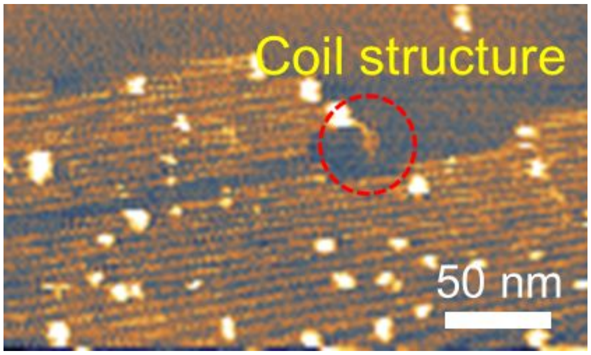

Figure S10. Topographic image of pSMAs with coil nanostructure at one end indicated by a red dashed circle.

Dormant state to activated state of pSMAs HS-AFM observations revealed peptide desorption was taken place once nanocluster was removed (red circles), leading pSMAs changed from dormant to activated state.

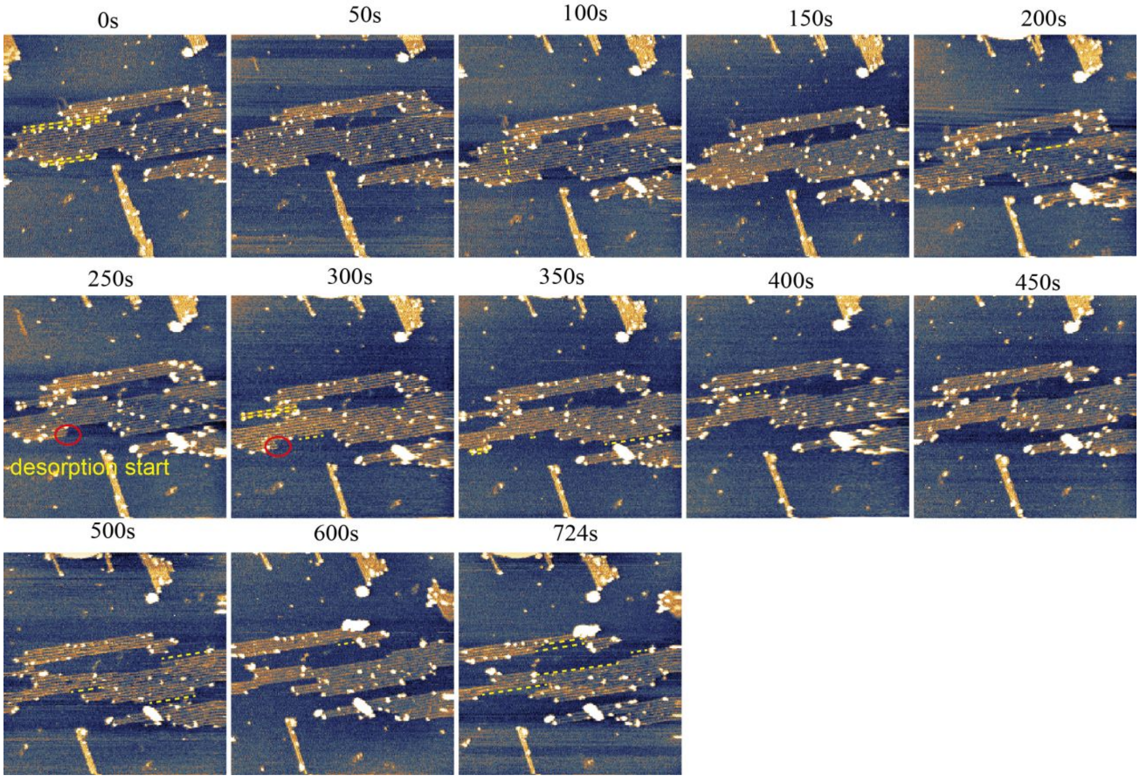

Figure S11. Dormant to activated state transition after removal of nanoclusters

Heights of pSMAs A height profile of self-assembled pSMAs

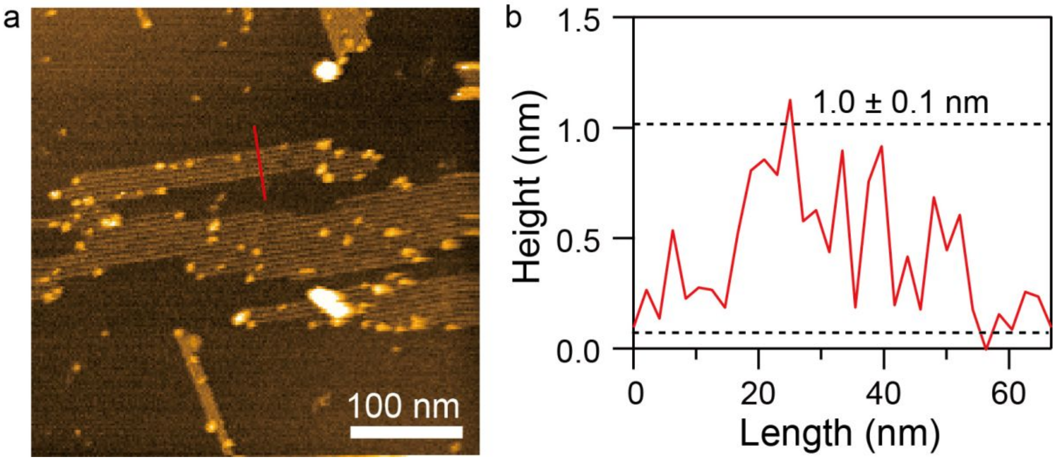

Figure S12. Morphology and Height of pSAMs in water condition. (a) A topographic image of pSMAs. (b) A line profile crossing pSMAs indicating a height of 1.0 ± 0.1 nm.

Phase transition (dormant state) during pSMAs desorption kinetics pSMAs imaged in an early time after water exchange by HS-AFM measurements.

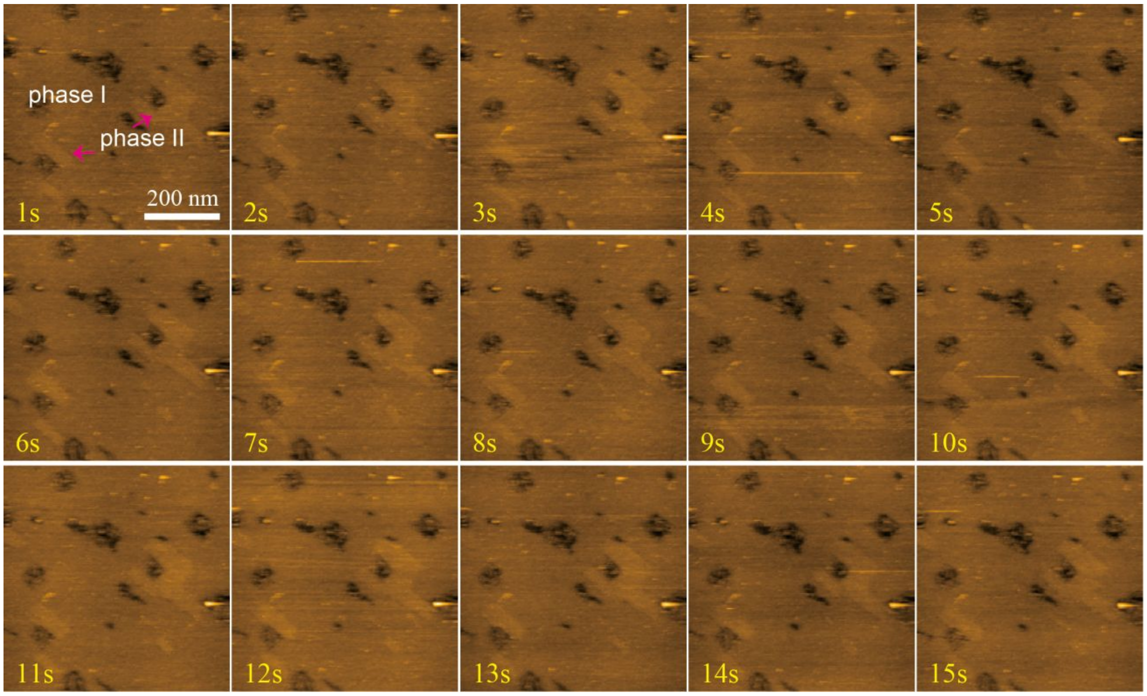

Figure S13. Dormant state of two phases from pSMAs in water incubation condition.

Desorption kinetics of phase II after undergoing Phase transition (activated state) *In-situ* HS-AFM further captured a desorption process of phase II after phase transition process corresponding to Figure 5.

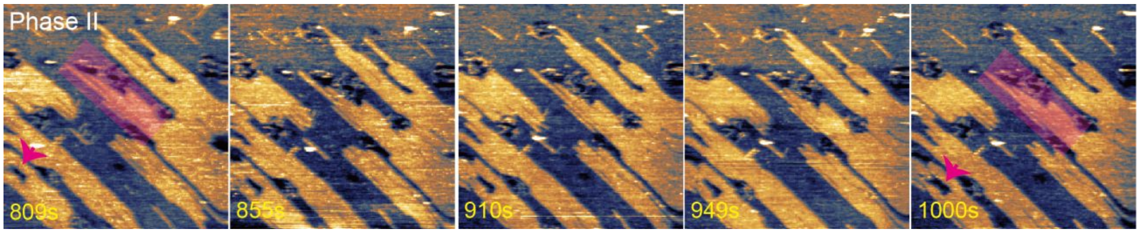

Figure S14. HS-AFM revealed a further desorption of phase II after undergoing a phase transition with phase I regarding Figure 5.

Binding affinity between peptides and MoS_2 AFM revealed the morphologies and coverage of peptides on solid surface with concentrations ranging from 0.1 to 5.0 μM. According to the Langmuir-Blodgett model, we plot the peptide coverage versus peptide concentrations. A binding constant of 1.03 μM^{-1} was obtained.

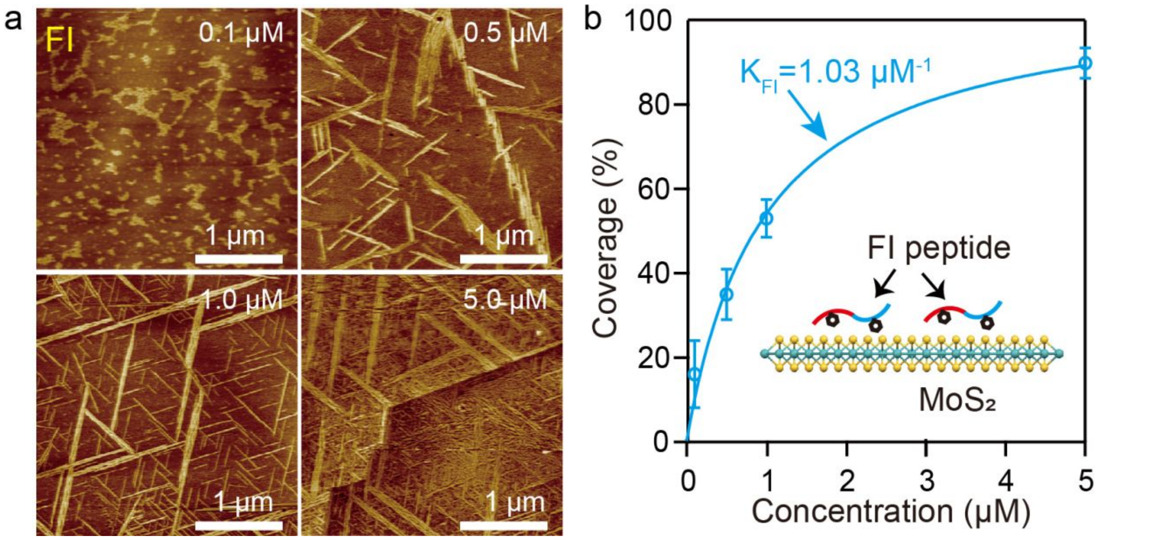

Figure S15 Binding affinity of peptides on MoS_2 surface. (a) Topographic images of self-assembled peptides on bulk MoS_2 with concentrations from 0.1 to 5.0 μM. (b) Binding affinity of FI peptides on bulk MoS_2 *via* a fitting of surface coverage and peptide concentrations based on the Langmuir-Blodgett model.

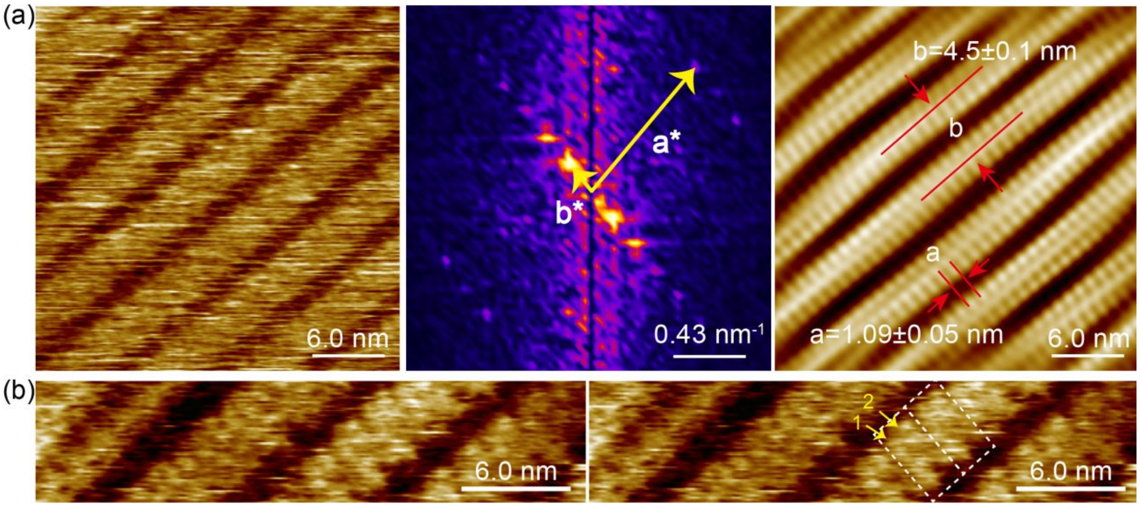

Figure S16 High resolution AFM image showing molecular-scale structure of FI peptides. (a) Topographic image revealing lattice parameters of unit cell of self-assembled FI peptides with a= 4.5 ± 0.1 and b= 1.09 ±0.05 nm. (b) High resolution showing a dimer structure along nanowire direction.

Desorption behaviors from two phases of FI peptides on HOPG Two phases have an intersect angle of 90 degrees. The eye-guided image showed the distribution of two phases.

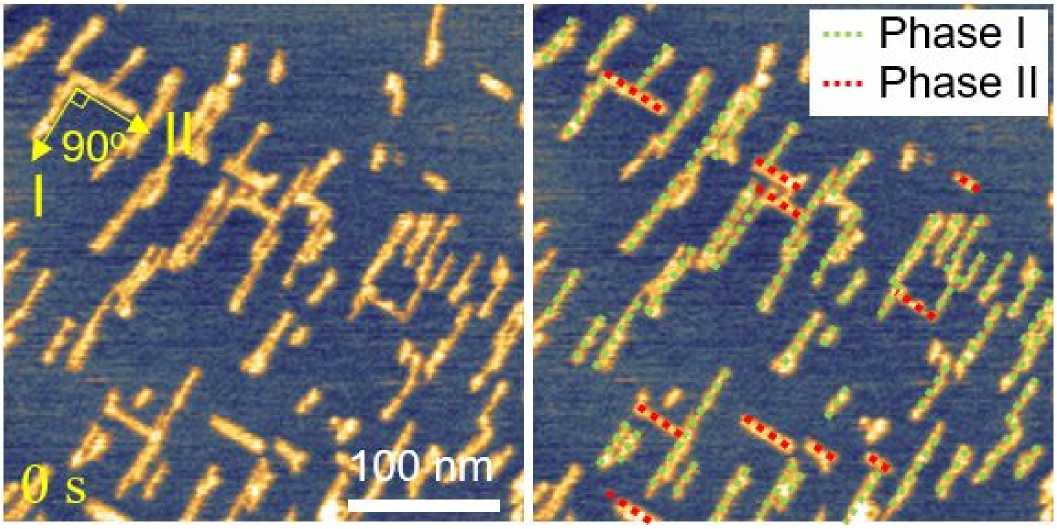

Figure S17 Two phases of FI peptides on HOPG surfaces observed by HS-AFM imaging

Table S1 Quantifying the correlation between single nanowire length change and time

NW3		-ln(-ln(L/L0))	t	ln(t-t0)
L0	87.9518		37	
L1	86.747	4.283587	38	0
L2	85.5422	3.583471	39	0.693147
L3	84.3373	3.170926	40	1.098612
L4	84.3373		41	
L5	84.3373		42	
L6	84.3373		43	
L7	83.1325	2.876116	44	1.94591
L8	80.7229	2.456125	45	2.079442
L9	80.7229		46	
L10	80.7229		47	
L11	78.3133	2.153549	48	2.397895
L12	75.9036	1.918016	49	2.484907
L13	75.9036		50	
L14	75.9036		51	
L15	74.6988	1.812014	52	2.70805

Table S2 Quantifying the correlation between single nanowire length change and time

NW6		-ln(-ln(L/L0))	t	ln(t-t0)
L0	91.5663		41	
L1	85.5422	2.687461	42	0
L2	78.3133	1.855685	43	0.693147
L3	72.2892	1.442278	44	1.098612
L4	61.4458	0.919025	45	1.386294
L5	53.012	0.604139	46	1.609438
L6	45.7831	0.366511	47	1.791759
L7	40.9639	0.217693	48	1.94591
L8	37.3494	0.108982	49	2.079442
L9	22.8916	-0.32663	50	2.197225
L10	12.0482	-0.70712	51	2.302585
L11	9.63855	-0.8115	52	2.397895

Table S3 Quantifying the correlation between peptide coverage change and time

Coverage		θ_t/θ_0	$-\ln(-\ln(\theta_t/\theta_0))$	$\Delta t(s)$	$\ln(\Delta t)$
θ_0	97.2			0	
θ_1	96.15	0.989198	4.522599	30	3.401197
θ_2	96.15	0.989198	4.5	50	3.912023
θ_3	95	0.977366	3.776877	122	4.805135
θ_4	92.1	0.947531	2.920706	207	5.333086
θ_5	80.8	0.831276	1.688517	492	6.198479
θ_6	87	0.895062	2.205104	538	6.287859
θ_7	81.2	0.835391	1.715602	559	6.326149
θ_8	67.8	0.697531	1.021073	594	6.386879
θ_9	57.2	0.588477	0.634468	628	6.44254
θ_{10}	50.4	0.518519	0.420408	677	6.517671
θ_{11}	48.9	0.503086	0.37543	708	6.562444
θ_{12}	48.4	0.497942	0.36058	745	6.613384
θ_{13}	46.8	0.481481	0.313494	788	6.669498
θ_{14}	51.1	0.52572	0.441632	809	6.695799
θ_{15}	50.9	0.523663	0.435553	855	6.751101
θ_{16}	48.5	0.498971	0.363545	910	6.813445
θ_{17}	48.2	0.495885	0.354661	949	6.855409
