# Supplementary material for: Deciphering Desorption Pathways and Mechanisms of Peptide Supramolecular Structures Thermodynamically and Kinetically by High-Speed AFM
Source: ACS Cent Sci. 2025 Apr 2;11(5):672–82. doi: 10.1021/acscentsci.5c00215 (PMC12123461; doi:10.1021/acscentsci.5c00215)
Supplement: Supplementary file 2 [file oc5c00215_si_002.pdf]

oc-2025-00215d.R1

Name: Peer Review Information for "Deciphering Desorption Pathways and Mechanism of Peptide Supramolecular Structures Thermodynamically and Kinetically by High-Speed AFM"

First Round of Reviewer Comments

Reviewer: 1

Comments to the Author

The authors used in situ high-resolution and high-speed AFM to study the desorption processes of peptide at vdW-liquid interfaces. Although studying biomolecular desorption from solid-liquid interfaces is essential to leading applications, the current manuscript has several critical questions to address.

1. I am not sure if the results of varying desorption temperature fit with the rest of the manuscript well. I assume all experiments in Fig. 3-7 were done at the same temperature. In addition, Fig. 2A shows that all the AFM experiments in Fig. 2 were done in the dehydrated state after a particular incubation time in water. If the authors did what they indicated in the scheme (Fig. 2a), how do you evaluate the dehydration factor in the proposed model?

2. In addition, the evidence of peptide diffusion to explain why the coverage of 45 degree is more than 35 degree is missing. In addition to coverage, the assembly order of 45 degree is also better than 35 degree. Peptide-peptide interaction and peptide-MoS_2 interaction both determine the assembly order. Can the ‘diffusion’ idea explain that?

3. Fig. 2f shows that the heights of peptide assembly are also temperature-dependent. Is that due to the peptide pack change? If so, the formula used in Fig 2 may not be valid anymore. How about the molecular packs in the rest of the work?

4. Again, what was the temperature in Fig. 3-7? How did you choose it, but not the rest of the two in Fig. 2?

5. Fig. 5 shows two phases. What was the phase studied in Fig. 3 and 4? If the studied phase in Fig. 3 and Fig. 4 was the metastable phase I, then the fine study of phase II might be more important.

6. What are the molecular interactions/packs in phase I and phase II? What are the interactions between the phases and MoS_2 lattice? Which determines the stability and desorption processes of the two phases?

7. The 'stop' mechanism proposed in Fig. 4 may not explain the gap stops in Fig. 3.

8. Apparently, the molecular interactions within a peptide row and between rows are different, so the bead mode in Fig. 4 is inaccurate. V_a and V_b should be different.

9. Fig. 6 does not include the binding affinity between peptide and MoS_2, so it does not explain the transition between phases I and II.

10. Does the desorption on HOPG also involve two phases?

11. Last, I wonder how the authors defined thermodynamic desorption and kinetic desorption. In Fig. 3-7, the authors studied the desorption dynamics. However, I am unsure which kinetic process/pathway was studied.

Reviewer: 2

Comments to the Author

In an impressive set of experiments, the authors investigate the desorption pathways and mechanism of peptide supramolecular structures, from a thermodynamic point of view, as well as a kinetics point of view, the latter using high-speed AFM. The authors established distinct desorption pathways, investigated in detail.

I only have one question and a remark

1) as far as the high-speed AFM measurements are concerned. To what extent are the dynamics / kinetics potentially affected by the scanning rate and scanning force?

2) In the outlook, the authors refer to the relevance of their study on drug effect on disassembly of amyloid protein fibrils in neurodegenerative diseases. However, I would like to see some arguments to substantiate this reasoning.

Author's Response to Peer Review Comments:

Senior Editor *ACS Central Science*

We thank the reviewers for their comprehensive review of our manuscript, along with their constructive comments and suggestions. We also appreciate the editor for great efforts and time on handing our manuscript, and providing us a valuable chance for resubmission after major revision.

Our detailed, point-by-point responses to each of the reviewer's comments are given below. We have incorporated corresponding revisions into the manuscript with additional texts and explanations, as well as updated supplementary Figures. The reviewers have made suggestions for further research. Also, there are other comments, e.g., Reviewer 1 “Again, what was the temperature in Fig. 3-7? How did you choose it, but not the rest of the two in Fig. 2” or “Fig. 5 shows two phases? What was the phase studied in Fig. 3 and 4? If the studied phase in Fig. 3 and Fig. 4 was the metastable phase I, then the fine study of phase II might be more important.” and Reviewer 2 “as far as the high-speed AFM measurements are concerned. To what extent are the dynamics / kinetics potentially affected by the scanning rate and scanning force?” and so on. These comments and suggestions are very useful and point to the need for further comprehensive research to fill in the gaps in the manuscript. We have provided more explanations based on the reviewers' suggestions to support the findings already addressed in our manuscript.

We believe that our manuscript has been considerably improved due to these revisions, and we hope that our revised manuscript has potential to be acceptable for publication in *ACS Central Science*. We thank you once again

for your consideration of our work and for inviting us to resubmit the revised manuscript. We look forward to hearing from you.

Formatting Needs:

Abstract: Please make sure the word count of your Abstract does not exceed 200 words.

Response: We thank the editor for the comment. We have confirmed the word count of the Abstract does not exceed 200 words.

Synopsis: ACS Central Science requires a brief synopsis. The synopsis should be no more than 200 characters (including spaces) and should reasonably correlate with the Table of Contents (TOC) graphic. The synopsis is intended to explain the importance of the article to a broader readership across the sciences. Please place your synopsis in the manuscript file after the TOC graphic.

Response: Thank you very much for the Editors' comments. We have added the Synopsis section after the TOC graphic in our revised manuscript.

Reviewer(s)' Comments to Author:

Reviewer: 1

Q1: Recommendation: Reconsider after major revisions noted.

Response: We thank the reviewer for their valuable and insightful comments, helping us to improve the quality of our paper. We have made significant modifications based on the reviewer's suggestive comments.

Q2: Comments:

The authors used in situ high-resolution and high-speed AFM to study the desorption processes of peptide at vdW-liquid interfaces. Although studying biomolecular desorption from solid-liquid interfaces is essential to leading applications, the current manuscript has several critical questions to address.

Response: We thank the reviewer for comprehensive understanding of our work. More importantly, we appreciate the reviewer for providing many essential comments. These comments have made our manuscript more attractive for potential readership.

Q3: 1. I am not sure if the results of varying desorption temperature fit with the rest of the manuscript well. I assume all experiments in Fig. 3-7 were done at the same temperature. In addition, Fig. 2A shows that all the AFM experiments in Fig. 2 were done in the dehydrated state after a particular incubation time in water. If the authors did what they indicated in the scheme (Fig. 2a), how do you evaluate the dehydration factor in the proposed model?

Response: We appreciate the reviewer for their insightful comments.

We first response to the reviewer's concern about the connection between varying desorption temperature and dynamical observations referring as the rest of the manuscript. As shown in Figure 2, peptide nanostructures at 25 degrees maintained a highly ordered phases for much longer time than those at 35 and 45 degrees. Peptide ordered phases allowed us to capture and record the detailed desorption features and pathways leading to unveiling its intrinsic desorption mechanism. That is why we selected 25 ^{o}C as a good candidate for dynamical observations as revealed in Fig. 3-7. The reviewer's assumption "I assume all experiments in Fig. 3-7 were done at the same temperature" is correct. We resolved those features, pathways and mechanisms at 25 ^{o}C.

As the reviewer commented correctly, all the AFM experiments in Fig. 2 were done in the dehydrated state after a particular incubation time in water. We believe the dehydration factor have effect on peptide desorption, such as peptide supramolecular arrays from rigid structure changing to loose structure. However, their structural ordering and crystallographic orientation would not change as shown (Figure RS1). The leftmost and

rightmost images are taken by in-situ and ex-situ AFM measurements at same position, respectively. After dehydration process, peptide nanostructure still remained a same orientation and kept same ordering, although it loses rigid structures. Therefore, we think the dehydration factor would not significantly change peptide desorption behaviors.

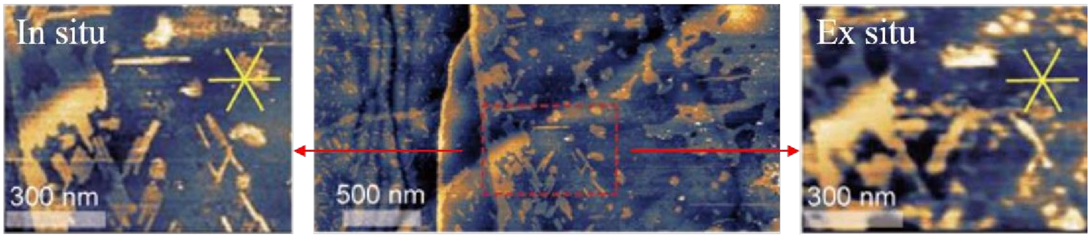

Figure RS1 AFM images of self-assembled peptides in dry and wet condition s from a same location. In- /ex-situ AFM images showed a same peptide orientations and ordering.

Q4: 2. In addition, the evidence of peptide diffusion to explain why the coverage of 45 degree is more than 35 degree is missing. In addition to coverage, the assembly order of 45 degree is also better than 35 degree. Peptide-peptide interaction and peptide-MoS_2 interaction both determine the assembly order. Can the 'diffusion' idea explain that?

Response: We thank the reviewer for the valuable comments.

We apologize for an inadequate explanation about the temperature effect on the peptide coverage changes. Following a thermodynamic equation (Eq. 1 in main text), a high temperature can lead to a fast desorption rate of molecules. In principle, peptide coverage at 45 °C should be lower than that at 35 °C. While temperature also played a significant role in promoting a fast diffusion of peptides on solid surfaces, followed by a rearrangement of desorbed molecules leading to the newly formed peptide nanowires. Therefore, a tradeoff between peptide desorption rates and diffusion rates can determine the final peptide coverage on solid surface. This could explain why the coverage of peptide at 45 °C is more than that at 35 °C.

According to the reviewer's comments on "Peptide-peptide interaction and peptide-MoS_2 interaction both determine the assembly order". This is absolutely correct. The balance between interpeptide interaction and peptide-MoS_2 interaction are also the key to keeping peptide structural ordering. Temperature changes can also affect these two interactions. As observed by Figures 2c-d, three-fold symmetry of peptide nanostructures was partially destroyed at 35 and 45 degrees at a long water incubation time. It indicates that the

temperature affects the interpeptides and peptide-MoS_2 interactions. The diffusion idea may not directly reflect the changes in structural ordering. However, as correctly pointed out by the reviewer, the structural ordering of peptides at 45 degrees is higher than that at 35 degrees. It may provide an indirect clue that the rearrangement of desorbed peptides still partially followed a certain crystallographic orientation on MoS_2 *via* a "Lattice matching" mechanism as widely found (Y Ayhan M Sarikaya et al., 2024, *Small*). Therefore, we thank the reviewer for providing this valuable comment, helping to explain the structural ordering from the interactions point of view.

Therefore, following the good suggestions by the reviewer, we have rewritten the sentences to address the temperature effects both on the coverage changes and structural ordering in our revised manuscript marked by yellow.

Q5: 3. Fig. 2f shows that the heights of peptide assembly are also temperature-dependent. Is that due to the peptide pack change? If so, the formula used in Fig 2 may not be valid anymore. How about the molecular packs in the rest of the work?

Response: We thank for the reviewer's comprehensive comments.

Yes, we think temperature changes the peptide pack from rigid folded nanostructures with well-organization towards gradually loosely bound nanostructures leading to the conformational changes as revealed by heights. The results of height changes observed in Figure 2f correspond to the same temperature, a changed parameter is incubation time. Therefore, the formula used in Fig. 2 is valid.

According to the second comments on "How about the molecular packs in the rest of the work?", our response is that the molecular packs were also changed. Some typical observations (Figs. 3b-c, Fig. 4c) showed dots-like structures or coiled structures at the nanowire edges, significantly differing from the well-organized nanowire structures. It indicates that the molecular packs have already changed their conformations.

Q6: 4. Again, what was the temperature in Fig. 3-7? How did you choose it, but not the rest of the two in Fig. 2?

Response: We thank the reviewer for the important comments.

The temperature in Fig. 3-7 is 25 °C. We selected 25 °C as a candidate temperature for dynamic observations because peptide nanostructures can maintain a highly ordered phase for a long time in contrast to the other two temperatures as revealed by Figure 2. Such ordered structure in a long-term allowed us to capture and record the detailed features and pathways of peptide desorption behaviors. Therefore, we choose this 25 °C for further kinetic studies.

Therefore, following the reviewer's comments, we clearly state the temperature condition in Figs. 3-7 and address why we choose the 25 °C for HS-AFM imaging in our revised manuscript.

Q7: 5. Fig. 5 shows two phases. What was the phase studied in Fig. 3 and 4? If the studied phase in Fig. 3 and Fig. 4 was the metastable phase I, then the fine study of phase II might be more important.

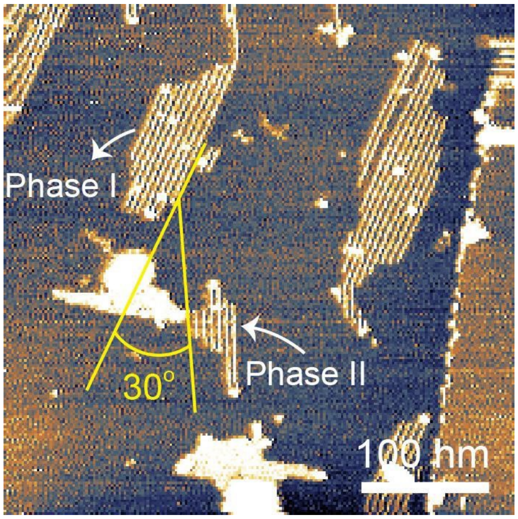

Figure RS2 A topographic image showing both phase s I and II of pep tide NWs during desorption process.

Response: We appreciate the reviewer for the insightful comments.

Indeed, both phases I and II were studied in Figs. 3 and 4. For example, a topographic image revealed by HS-AFM (Fig. RS2) corresponding to the panel (b) of Figure 4 in the main text. Nanowires (NWs) from both phases showed an intersect angle of 30 degrees, which is the same angle offset as shown in Fig. 5. Dynamical observations by HS-AFM measurements showed that both phases during desorption process exhibited a similar desorption features and pathways.

According to the reviewers' comment "If the studied phase in Fig. 3 and Fig. 4 was the metastable phase I, then the fine study of phase II might be more important.", this is a very interesting idea. For the FI peptides used in this work, since both phases showed similar desorption features, the studying on any of two phases would be important. While we

would like to examine the above reviewer's assumptions by designing and characterizing other peptide sequences such as graphite-binding peptides or fibroin-like silk peptides in our future work.

Therefore, we have added one sentence to clearly claim that desorption features and pathways were observed for both phases I and II in both Figures 3 and 4.

Q8: 6. What are the molecular interactions/packs in phase I and phase II? What are the interactions between the phases and MoS2 lattice? Which determines the stability and desorption processes of the two phases?

Response: We thank the reviewer for the suggestive comments.

First, the FI peptide sequence contained two positively charged Lys (K) amino acids and two negatively charged Glu (E) amino acids (see Figure 1a). Interpeptide could interact with each other by electrostatic interactions between K and E amino acids to form a dimmer-like unit (Figure RS3). Second, the π electron of Phe (F) could interact with the MoS_2 lattice leading to peptides anchor onto MoS_2 surface as reported in literature (S. Cetinel et al., *Sci. Rep.*, 2018, 8). Besides, the vdW interactions or hydrophobic interactions between peptides and MoS_2 lattice could facilitate peptides binding to MoS_2 surfaces.

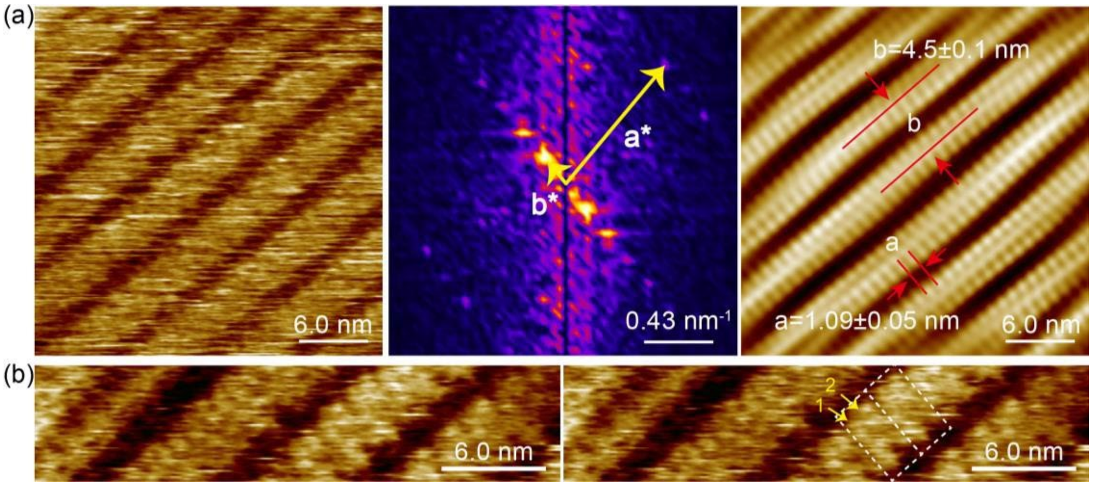

Figure RS3 High resolution AFM image showingmolecular-scale structure of FI petpides. (a) Topographic image revealing lattice parameters of unit cell of self -assembled FI peptides with a= 4.5 ± 0.1 and b= 1.09 ±0.05 nm. (b) High resolution showing a dimer structure along nanowire direction.

Both intermolecular interactions along NWs direction and vertical to NWs direction, as well as molecules-MoS_2 interactions are important for determining the stability and desorption process. From our HS-AFM observations, desorption behavior could be laughed from terminals or middle part of NWs, where peptides need to overcome the energy barrier from intermolecular interaction and peptide-substrate interactions. The formation of highly ordered NWs structures with different crystallographic orientations in phases I and II is modulated by MoS_2 lattice following a "Lattice-matching" mechanism (A Yurtsever, M Sarikaya et al., *ACS Nano*, 2023). The intrinsic mechanism could affect the folded structures of self-assembled peptides leading to changes in crystallographic orientations. Also, both phases of peptide NWs have different energy landscape. During the water incubation process, the metaphase would either commence to desorption or transform to stable phase via phase transition, followed by second desorption

Therefore, we have added the Figure RS3 into Supporting information as Figure S16.

Q9: 7. The 'stop' mechanism proposed in Fig. 4 may not explain the gap stops in Fig. 3.

Response: We thank the reviewer for the comments.

In Figure RS4 from t=41 to 45 sec corresponding to Figure 3b, we also detected a "stop" mechanism as proposed in Fig. 4. Therefore, these results from Figs. 3 and 4 are highly consistent.

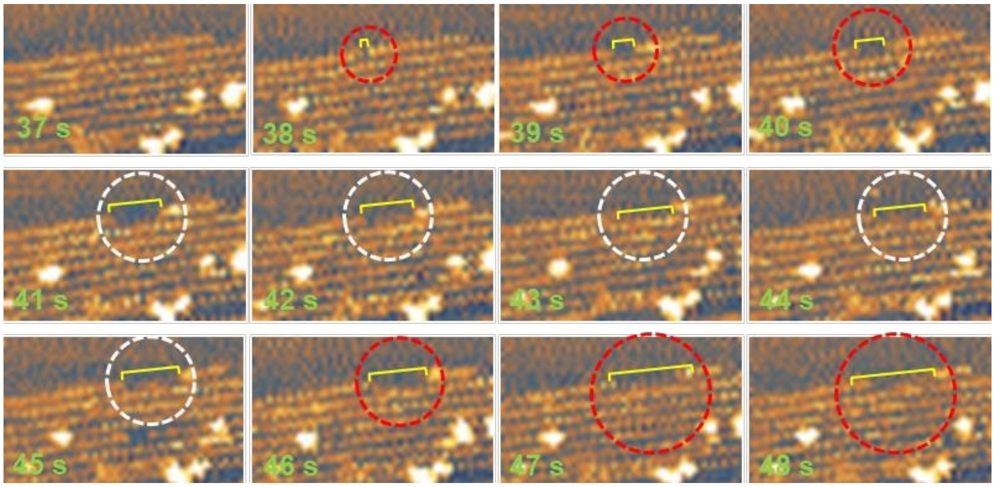

Figure RS4. HS-AFM imaging to reveal detailed features in dynamics, which corresponds to the Figure 3b. Red and white circles means nanogap enlargement and stop.

Q10: 8. Apparently, the molecular interactions within a peptide row and between rows are different, so the bead mode in Fig. 4 is inaccurate. Va and Vb should be different.

Response: We thank the reviewer for the nice comments.

Following the reviewers' comments, we have redrawn a model as shown (Figure RS5), which allows us to differ Va from Vb. Therefore, we added this new model in Figure 4f.

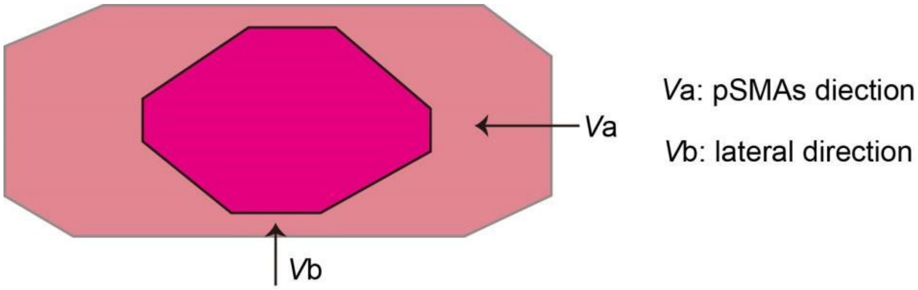

Figure RS5 A model of peptide desorption along NWs and vertical to NWs directions.

Q11: 9. Fig. 6 does not include the binding affinity between peptide and MoS_2, so it does not explain the transition between phases I and II.

Response: We appreciate the reviewer for the nice comment.

As commented by the reviewer, we examined the binding affinity between peptide and MoS_2 as shown (Figure RS6), where four different peptide concentrations from 0.1 to 5.0 μM were utilized and AFM measurements revealed their morphologies and coverages at each concentration. Following a classic protocol in calculating peptide binding affinity reported by Prof. Sarikaya group (Christopher R.So, M Sarikaya et al., *ACS Nano*, 2012), we plotted a relationship of peptide surface coverage versus peptide concentrations fitted by a classic Langmuir-Blodgett model (Figure RS6b). We obtained a binding affinity KFI equal to 1.03 μM^{-1}.

Therefore, we include this additional experimental results in supporting information as Figure S15. Also, in our revised manuscript, we provided the information of peptide binding affinity when discussed about the phase transition marked by yellow.

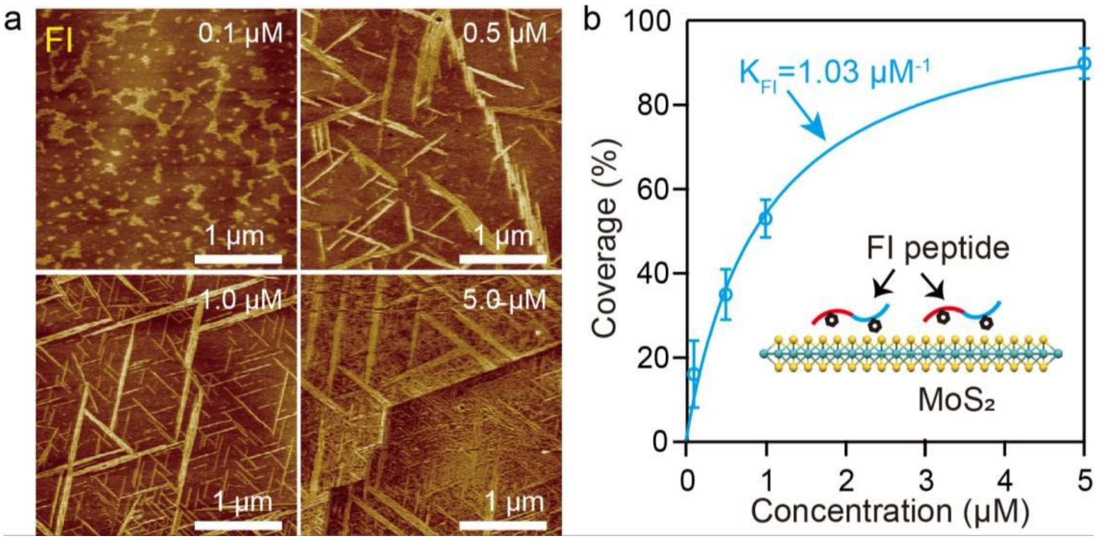

Figure RS6 Binding affinity of FI peptides on MoS_2 surface. (a) Topographic images of self-assembled peptides on bulk MoS_2 with peptide concentrations from 0.1 to 5.0 μM. (b) Binding affinity of FI peptides on bulk MoS_2 *via* a fitting of surface coverage and peptide concentrations based on the Langmuir-Blodgett model.

Q12: 10. Does the desorption on HOPG also involve two phases?

Response: We thank the reviewer for valuable comments.

Yes, the desorption on HOPG also involve two phases as shown (Figure RS7), corresponding to a topographic image at t=0s in Figure 7a. Two phases I and II have an intersect angle of 90 degrees. The eye-guided lines over the NWs in Figure RS7 (right) allows to distinguish them.

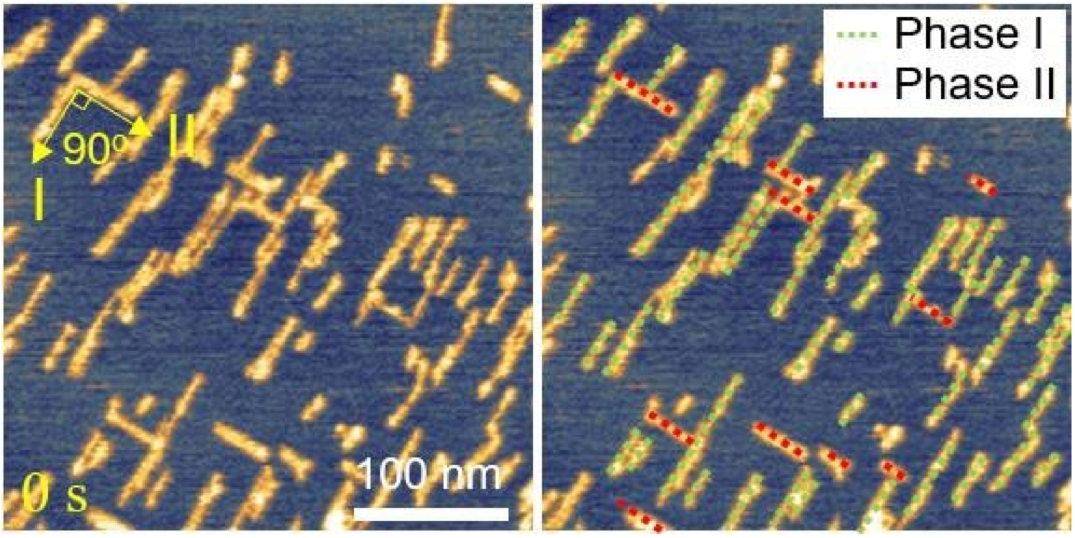

Figure RS7 Two phases of FI peptides on HOPG surfaces observed by HSAFM imaging

Therefore, following the reviewer's comment, we have added several sentences (marked by yellow) in the revised manuscript to mention the coexisted two phases on HOPG surface as well. And we added the analysis of Figure RS5 in Supporting Information as Figure S17.

Q13: 11. Last, I wonder how the authors defined thermodynamic desorption and kinetic desorption. In Fig. 3-7, the authors studied the desorption dynamics. However, I am unsure which kinetic process/pathway was studied.

Response: We thank the reviewer for the insightful comments.

We appreciate the reviewer asking a general while very important question about definition of thermodynamic desorption and kinetic desorption. In our understanding, Thermal desorption relates to use heat, or say temperature, to volatilize contaminants from a solid matrix (like soil or samples) for removal or analysis. Kinetic desorption refers to the time-dependent process where adsorbed atoms or molecules are released from a

surface into the surrounding vacuum or fluid after they gain enough energy to overcome the activation barrier and binding energy holding them to the surface.

In our work, we provided a variant of temperature to insight into how temperature change the morphologies, structural ordering and surface coverage of peptide nanowires (Figure 2). As correctly pointed out by the reviewer, this process also refers to a series of surface dynamics including peptide detachment, peptide diffusion, peptide reorganization. All these surface behaviors can be defined as kinetics as well. We cannot simply say this is thermodynamics or kinetics. However, we can select to focus on one specific aspect, for example, how temperature change peptide coverage? We establish the correlation between temperature and peptide number density. This process can be considered as thermodynamic desorption.

According to the comment "In Fig. 3-7, the authors studied the desorption dynamics. However, I am unsure which kinetic process/pathway was studied", we investigated the different desorption pathways as summarized in Figure 6. The HS-AFM observations allowed to detect (i) no disassembly in a short time, where active energy is necessary to induce the occurrence of pNWs desorption, (ii) asymmetrical desorption, that is, one end of NWs stop desorption while another end continue to disassembly. The desorption rate as a feature of dynamic process is quantitatively analyzed. Also, the desorption along NWs direction and vertical to NWs direction also be included in the dynamical observations. (iii) Bidirectional disassembly, both ends of pNWs start to disassembly while their desorption rates are not identical. (iv) Middle disassembly, rather than desorption taken place at both terminal of pNWs, desorption also observed to occur in the middle of pNWs. All the above pathways are monitored with time-dependent observations by HS-AFM measurements and pNWs showed a gradually shortening process, which indicated a kinetic process.

Again, we like the comments given by the reviewer. This comments can always remind us to consider the importance of thermodynamic and kinetic process as studying on peptide self-assembly or peptide disassembly on various solid surfaces such as HOPG, MoS_2, Boron nitride, Au, Glass, Mica and so on.

Reviewer: 2

Q1: Recommendation: Publish in ACS Central Science after minor revisions noted.

Response: We thank the reviewer for the positive comments on publishing this work in ACS Central Science after minor revisions.

Q2: Comments:
In an impressive set of experiments, the authors investigate the desorption pathways and mechanism of peptide supramolecular structures, from a thermodynamic point of view, as well as a kinetics point of view, the latter using high-speed AFM. The authors established distinct desorption pathways, investigated in detail. I only have one question and a remark

Response: We thank the reviewer for highlighting the importance and novelty of our work, and providing us valuable comments for further improving the quality of our work.

Q3: 1) as far as the high-speed AFM measurements are concerned. To what extent are the dynamics / kinetics potentially affected by the scanning rate and scanning force?

Response: We appreciate the reviewer for insightful comments.

Basically, three parameters during AFM measurements can have a large influence on the dynamics/kinetics including scanning rate, scanning force, and cantilever stiffness. Their effects depend on the material properties, interaction forces, and the timescales of the dynamic processes being studied.

According to scanning rate, a low scanning rate may not allow to capture a short momentary events, leading to an underestimation of kinetic rates. Oppositely, an excessively fast scan could make the feedback system lose regulation, causing imaging distortions or artifacts. Also, too fast scanning can disrupt equilibrium states, especially in soft or biological samples, leading to deviations from natural kinetics. Moreover, a rapid movement of tip may also introduce heating or vibration effects.

Regarding to scanning force, a higher tip force may perturb weak interactions, potentially altering molecular conformations or dissociation rates. Also, a higher scanning force can induce sample deformation leading to incorrect interpretations of dynamic processes.

Therefore, to overcome the effect from scanning rate and force, it is essential to optimize the scanning speed to capture the kinetics/dynamics without distorting them. Also, use a lowest possible force allowing to maintains a stable image. In our experiments, a soft cantilever was used to capture the peptide desorption dynamics/kinetics. Besides, a typical scanning rate of 0.5~1 s/f for HS-SICM measurements was used, which allows to obtain a reliable data.

Q4: 2) In the outlook, the authors refer to the relevance of their study on drug effect on disassembly of amyloid protein fibrils in neurodegenerative diseases. However, I would like to see some arguments to substantiate this reasoning.

Response: We thank the reviewer for the suggestive comments.

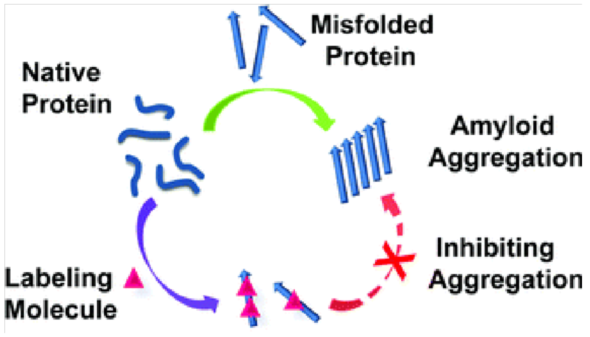

Figure RS1. Amyloid aggregation process.

As it is reported in literature (C Wang et al., Nanoscale, 2012, 4, 1895-1909), an aggregation process of misfolded proteins can induce neurodegenerative diseases (Figure RS1). While single native protein molecules have no such effect.

Thus, rather than studying on amyloid aggregation, many researchers have focused on a reversible process, that is, disassembly of amyloid aggregation by drugs.

The disassembly process of drug-induced amyloid fibrils refers to how strongly a drug binds to amyloid fibrils and whether it can dissociate after disrupting fibril structure, this process is similar to peptide nanowires desorption pathways in our studies. The binding sites, e.g., both ends of fibrils or in middle of fibrils, is important for drug molecules interacting to amyloid fibrils. Also, how long does it take for drug binding to amyloid fibrils until dissociate by drug molecules? and the dissociate rates at different local region of amyloids, homogeneous or heterogeneous. These features need to be captured by studying on dynamics and kinetics as HS-AFM revealed for peptide desorption in our work.

Besides, peptide desorption refers to how water molecules binding and interacting with peptide composed of nanowires and make peptide molecule disassociate from nanowires. This process is analog to amyloid fibril disassembly, where some drugs may replace stabilizing molecules within amyloid fibrils, weakening their structure (Y Soeda et al., *J Alzheimer's Dis*., 2019).

Also, peptide desorption studies help determine if a drug facilitates the release of monomers or smaller oligomers from fibrils, leading to degradation (R Malik et al., *Springer Protocols*, 2018). In our observations, nanodots and random coiled structures during peptide desorption process were captured, where the desorption details also can provide a good understanding of how drug facilitates the amyloid fibrils to disassembled into monomers or oligomers.

Last, environmental factor (pH, ionic strength, and temperature) is also essential to evaluate the disassembly of amyloid fibrils by affecting drug efficacy. Some fibrildisrupting drugs work better under physiological conditions, while others may require cofactors to enhance desorption. Here, in peptide desorption studies, we investigated how temperature can affect the morphologies, structural ordering and peptide number density. This point may also provide insight into environmental factor affecting disassembly of amyloid fibrils.

Considering these similarities, we, therefore, mentioned the relevance of our study on drug effect on disassembly of amyloid protein fibrils in neurodegenerative diseases in the background sections. This current work may provide some basis for further investigating drug effect on amyloid fibrils. In the future, we would like to real-time imaging the drug effects on amyloid fibrils by HS-AFM measurements.
